# Spin‐Controlled Binding of Carbon Dioxide by an Iron Center: Insights from Ultrafast Mid‐Infrared Spectroscopy

**DOI:** 10.1002/anie.202012739

**Published:** 2020-11-27

**Authors:** Steffen Straub, Peter Vöhringer

**Affiliations:** ^1^ Rheinische Friedrich-Wilhelms-Universität Institut für Physikalische und Theoretische Chemie Wegelerstrasse 12 53115 Bonn Germany

**Keywords:** carbon dioxide complexes, CO_2_-activation, femtochemistry, iron, time-resolved infrared spectroscopy

## Abstract

The influence of the spin on the mode of binding between carbon dioxide (CO_2_) and a transition‐metal (TM) center is an entirely open question. Herein, we use an iron(III) oxalato complex with nearly vanishing doublet–sextet gap, and its ultrafast photolysis, to generate TM‐CO_2_ bonding patterns and determine their structure in situ by femtosecond mid‐infrared spectroscopy. The formation of the nascent TM‐CO_2_ species according to [L_4_Fe^III^(C_2_O_4_)]^+^ + *hν* → [L_4_Fe(CO_2_)]^+^ + CO_2_, with L_4_=cyclam, is evidenced by the coincident appearance of the characteristic asymmetric stretching absorption of the CO_2_‐ligand between 1600 cm^−1^ and 1800 cm^−1^ and that of the free CO_2_‐co‐fragment near 2337 cm^−1^. On the high‐spin surface (*S*=5/2), the product complex features a bent carbon dioxide radical anion ligand that is O‐“end‐on”‐bound to the metal. In contrast, on the intermediate‐spin and low‐spin surfaces, the product exhibits a “side‐on”‐bound, bent carbon dioxide ligand that has either a partial open‐shell (for *S*=3/2) or fully closed‐shell character (for *S*=1/2).

## Graphical abstract

Snapshots from femtosecond mid‐infrared laser spectroscopy reveal spin‐dependent formation of different binding modes of carbon dioxide to a transition‐metal center. The ultrafast excitation of an iron(III) oxalate precursor complex with ultraviolet pulses releases a neutral carbon dioxide molecule within 500 fs and generates a nascent iron‐CO_2_‐primary complex that isomerizes on a picosecond time scale.

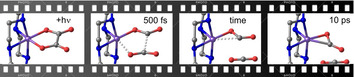

## Introduction

The binding of carbon dioxide to transition metals (TM), preferably earth‐abundant TMs like iron, is a captivating and highly challenging topic of Coordination Chemistry with important technological implications for designing sustainable circular economies that exploit anthropogenic greenhouse gas emissions.^[1]^ The key chemical step is the conversion of CO_2_ into its reductively activated form, the CO_2_^.−^ radical anion, which requires an energy of roughly 40 kJ mol^−1^ in the gas phase.^[2]^ However, in aqueous solution, the energetics are actually reversed due to the solvation free energy of the open‐shell anion.^[3]^ A value of −1.90 eV is currently accepted for the reduction potential of CO_2_,^[4]^ which translates into an adiabatic electron affinity in water of about 2.5 eV, or equivalently, 240 kJ mol^−1^.^[5]^ This suggests that in condensed phase environments, a CO_2_^.−^ radical anion will be most useful for chemical conversions before the inevitable process of solvation has significantly tamed its innate chemical reactivity.

The binding to a TM on the other hand may allow for transferring electron density to the neutral CO_2_, thereby converting it into its reductively activated form and, at the same time, preventing it ‐at least temporarily‐ from being released into the stabilizing solvent environment. Thus, the binding to a TM may open up a time window that is sufficiently long for a chemical substrate to approach the TM‐CO_2_ complex and to engage in a desired chemical transformation with the carbonaceous triatomic ligand.

**Figure 1:**
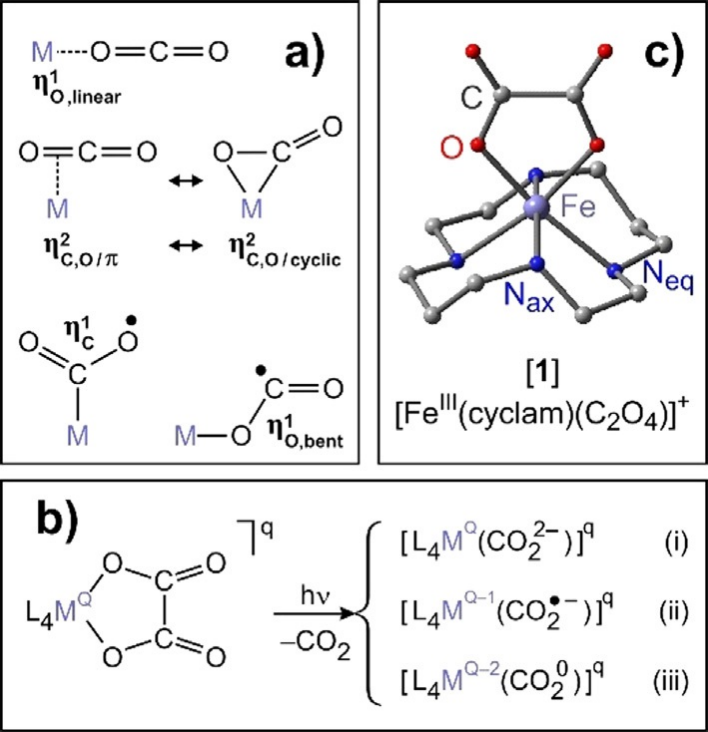
a) TM‐CO_2_ bonding patterns. Top: linear‐O‐“end‐on”, middle: “side‐on” with the two mesomeric structures, π‐complex and metallaoxacyclopropanone. Bottom left: C‐bound “Y‐on” and bottom right: bent‐O‐“end‐on”. b) Photoinduced CO_2_‐release from TM‐oxalato precursor complexes. L_4_ represents additional ligands; for example, a tetradentate ancillary ligand. c) DFT‐optimized molecular structure of [**1**].

To explore such concepts further, a thorough understanding of the electronic, structural, and dynamical principles that govern CO_2_‐TM binding is of paramount importance.[^1b^, ^6^] CO_2_ can actually bind to TMs in a variety of structural motifs,[^6a^, ^7^] the most prominent ones are shown schematically in Figure 1 a. The different motifs can involve (i) the binding of a linear ligand via a terminal oxygen atom,^[8]^ that is, the O‐“end‐on” mode, $\eta_{O,linear}^{1}$ , (ii) the binding of a bent ligand forming either a π‐complex or a metallaoxacyclopropanone,^[9]^ that is, the “side‐on”‐mode, $\eta_{CO}^{2}$ , and (iii) the binding of a bent ligand via the central carbon atom,^[10]^ or in short, the “Y‐on”‐mode, $\eta_{C}^{1}$ . A fourth binding mode, $\eta_{O,bent}^{1}$ , has also been discovered very recently,^[11]^ which involves again the binding via a terminal oxygen atom but in a bent geometry.

When attached to a metal, M, the oxalate anion, C_2_O_4_^2−^, becomes a photolabile ligand that can dissociate upon optical excitation into a neutral CO_2_‐molecule and a CO_2_‐dianion (carbonite^[12]^); that is, C_2_O_4_^2−^ + *hν* → CO_2_^2−^ + CO_2_. This heterolytic C−C bond fission causes the neutral fragment to depart from the ligand sphere of TM‐oxalato complexes—a well‐known photochemical reaction, which constitutes the fundamental basis of optical dosimetry with the ferrioxalate standard.^[13]^ At the same time, the dianionic fragment is retained by the TM‐center as a redox non‐innocent ligand as shown in Figure 1 b. The oxidation state, Q, of the metal is only preserved if the CO_2_‐ligand binds as a genuine dianion, (CO_2_^2−^), (cf. pathway (i)). A one‐electron transfer from the ligand to the metal generates the triatomic radical anion ligand, (CO_2_^.−^), and reduces the metal to the oxidation state, Q‐1 (pathway (ii). Similarly, the two‐electron reduction of the metal to Q‐2 is also feasible and involves the neutral ligand, (CO_2_^0^), (pathway (iii)).

Since CO_2_ is expelled as a closed‐shell neutral molecule, there are two parameters, which have to be conserved in all three pathways (i)–(iii); namely, the outer charge, q, and the overall spin, S. In other words, the TM‐CO_2_‐product in Figure 1 b must inherit both, q and S, from the precursor unless intersystem crossings and/or electron detachments precede the actual bond breakages. If the triatomic ligand emerges from the photoreaction as a purely closed‐shell moiety via the pathways (i) and (iii), then the products are likely to adopt the $\eta_{CO}^{2}$ and $\eta_{O,linear}^{1}$ binding modes. In contrast, an open‐shell carbonaceous ligand generated via pathway (ii) coordinates most likely in the $\eta_{C}^{1}$ and $\eta_{O,bent}^{1}$ ‐motifs.

We have recently utilized ferrioxalate and its photochemistry as a model system for studying the dynamics and the vibrational spectroscopy of nascent CO_2_‐complexes of iron in liquid solution.[^11^, ^14^] Transferring the concept of Figure 1 b onto ferrioxalate, we have M=Fe, Q=+III, L_4_=(C_2_O_4_)_2_, and q=−3. Importantly, the rather weak ligand field enforced by the two additional oxalate ligands results in a high‐spin (d^5^, *S*=5/2) electron configuration at the metal. Our experiments gave very clear evidence that in this particular system, only the pathway (ii) is open. The resultant product featured the $\eta_{O,bent}^{1}$ ‐mode with the bent (CO_2_^.−^)‐ligand (*S*=1/2) ferromagnetically coupled to a high‐spin (d^6^, *S*=2) iron(II) center to conserve the parent's multiplicity. Since neither the “side‐on” nor the linear‐O‐“end‐on” modes were observed, the apparent product distribution must be a direct consequence of spin conservation during the photo‐transformation and the spin‐state energetics of the photoproduct.^[15]^

Then, the question naturally arises whether or not it is possible to steer the photochemistry towards products exhibiting closed‐shell forms of the triatomic carbonaceous ligand and in particular, the $\eta_{CO}^{2}$ binding mode. Intuitively, fully complementary experiments on a low‐spin TM‐oxalato system are desired to test such a strategy. Starting from ferrioxalate and guided by the spectrochemical series, we have modified L_4_ and replaced two oxalate ligands with the neutral tetradentate auxiliary ligand, cyclam (L_4_=1,4,8,11‐tetraazacyclotetradecane). Due to the four secondary amine moieties, the resultant cationic (q=1), ferric (Q=III) precursor, [Fe(cyclam)(C_2_O_4_)]^+^ ([**1**]), as well as its photochemical products, [Fe(cyclam)(CO_2_)]^+^, should have a larger d‐orbital splitting than their L_4_=(C_2_O_4_)_2_ congeners. The resultant energetic stabilization of the low‐spin states relative to the high‐spin states should then affect the product distribution of CO_2_‐binding patterns in favor of the $\eta_{CO}^{2}$ ‐mode.

## Results and Discussion

**Figure 2:**
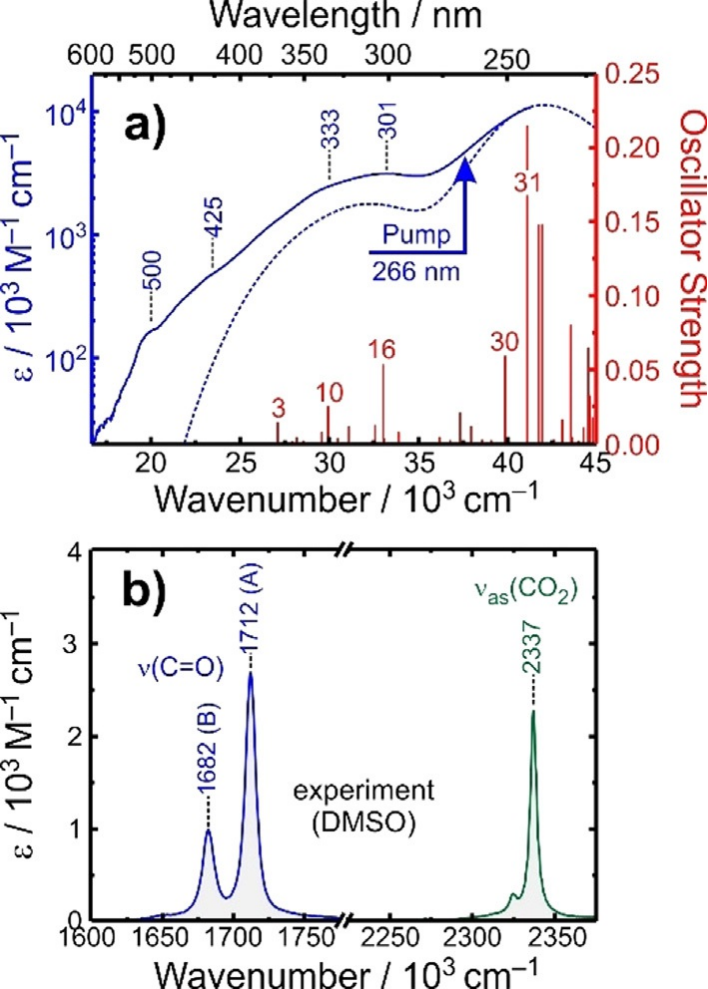
a) Experimental extinction coefficient, *ϵ*, of [**1**] in liquid DMSO solution at room temperature recorded in the UV/Vis‐spectral region. Solid blue (left ordinate): experimental, red sticks (right ordinate): theoretical oscillator strengths from TD‐DFT, dashed blue: TD‐DFT spectrum obtained by convoluting the stick spectrum with a Gaussian line shape having a full width at half maximum of 2000 cm^−1^. The arrow indicates the spectral position of the photolysis pulse, that is, 266 nm. Blue numbers specify spectral locations in nm, red numbers correspond to the indices of the most prominent TD‐DFT roots. b) Experimental extinction coefficient from a stationary FTIR‐spectrum of [**1**] (blue, left abscissa) and carbon dioxide (green, right abscissa), both in room temperature DMSO solution. Numbers indicate peak positions in cm^−1^.

The stationary electronic absorption spectrum of [**1**] in liquid dimethylsulfoxide (DMSO) solution is displayed in Figure 2 a. In the near‐UV region (*λ*≥250 nm), it consists of a highly characteristic band centered at 301 nm that exhibits a number of distinct shoulders at its low‐energy tail, most notably around 500 nm, 425 nm and 333 nm. Deeper into the ultraviolet region (*λ*≤250 nm), the onset of a very strong resonance is detected but owing to the limited transparency of the solvent, its exact peak location could not be determined.

Time‐dependent (TD) density‐functional theory (DFT, cf. supporting information, SI, Figure S1) calculations suggest that all of these features are due to a variety of different ligand‐to‐metal charge transfer transitions (LMCT) originating from the sextet state. Here, we report on the photolysis of [**1**] using laser pulses centered at 266 nm, that is, with light that is tuned to the two strongest LMCT resonances (cf. SI, Figure S2, roots 30 and 31), which are connected with shifts of charge density from the non‐bonding p‐orbitals of either the oxalate's O‐atoms or the cyclam‐N‐atoms into the iron‐d(z^2^) orbital. The ensuing photochemical dynamics were probed with femtosecond‐mid‐infrared (MIR)‐spectroscopy.

The stationary Fourier‐transform infrared (FTIR) spectrum in the carboxylate stretching region of [**1**] in room temperature DMSO solution is depicted in Figure 2 b. The oxalate ligand exhibits four CO‐bonds. Therefore, four CO‐stretching vibrations are expected and all of them should be IR‐active. The local CO‐motions couple to form two pairs of normal modes; namely the symmetric (or in‐phase) and antisymmetric (or out‐of‐phase) stretching vibrations of the inner C‐O groups ligating to the metal and the corresponding vibrations of the peripheral C=O groups. The former pair is usually observed between 1200 cm^−1^ and 1400 cm^−1^ (cf. SI), whereas the latter pair falls right into the spectral region that is shown in Figure 2 b. Thus, we attribute the stronger peak at 1712 cm^−1^ (denoted band A) to the in‐phase combination, ν_ss_(C=O), and the weaker peak at 1682 cm^−1^ (denoted band B) to the out‐of‐phase combination, ν_as_(C=O).

However, temperature‐dependent FTIR spectra indicate that this assignment may not be fully comprehensive. It turns out that in the temperature interval between 20 °C and 100 °C, both bands slightly decay and some additional red‐shifted IR‐activity grows in (cf. SI, Figure S3), thereby hinting at the existence of more than just a single species contributing to the spectrum. To follow up on this idea, solid‐state magnetometry was also carried out (see SI, Figure S4). The temperature‐dependent susceptibility is indeed indicative of a very broad thermally induced spin‐crossover (SCO) from low‐spin to high‐spin occurring at ≈213 K with Δ*H*_SCO_≈6 kJ mol^−1^ and Δ*S*_SCO_≈29 J mol^−1^ K^−1^. Yet, EPR‐spectroscopy in frozen DMSO at 18 K (see SI, Figure S5) suggests that the complex still adopts a sextet ground‐state in dilute solution. Taken all these data together, we can conclude that the introduction of the cyclam ligand has indeed affected the doublet‐sextet gap as we anticipated. Contrary to the solid state, however, the complex [**1**] still occupies a high‐spin ground‐state in liquid solution but there is evidence that the doublet state becomes thermally populated on raising the temperature.

The prediction of spin‐state energetics is a highly challenging task for DFT‐methods. Therefore, we use these experimental results in a next step to assess the reliability of a variety of DFT‐functionals in predicting the doublet‐sextet gap of [**1**] (cf. SI, Table S1). The pure functional, OPBE, which in the past was shown to be very reliable for such purposes,^[16]^ locates the sextet state at an energy of only 6.8 kJ mol^−1^ (568 cm^−1^) above the doublet state. In contrast, the presumably most accurate of all tested model chemistries, the double‐hybrid functional, B2PLYP,^[17]^ favors the sextet state but only by a mere 10.5 kJ mol^−1^ (or equivalently, 874 cm^−1^). Interestingly, Truhlar's hybrid‐meta functional, mPW1B95,^[18]^ returns a doublet‐sextet splitting of only 49 cm^−1^ (in favor of the sextet) and it thus predicts the high‐spin and low‐spin states to be energetically nearly fully degenerate. Therefore, we will use this functional in the remainder for interpreting the femtosecond MIR‐spectra following UV‐photolysis of [**1**] in DMSO solution. Complementary information obtained with the PBE0‐functional,^[19]^ which returns a doublet‐sextet gap that is in excellent agreement with that of B2PLYP, are given in the SI.

**Figure 3:**
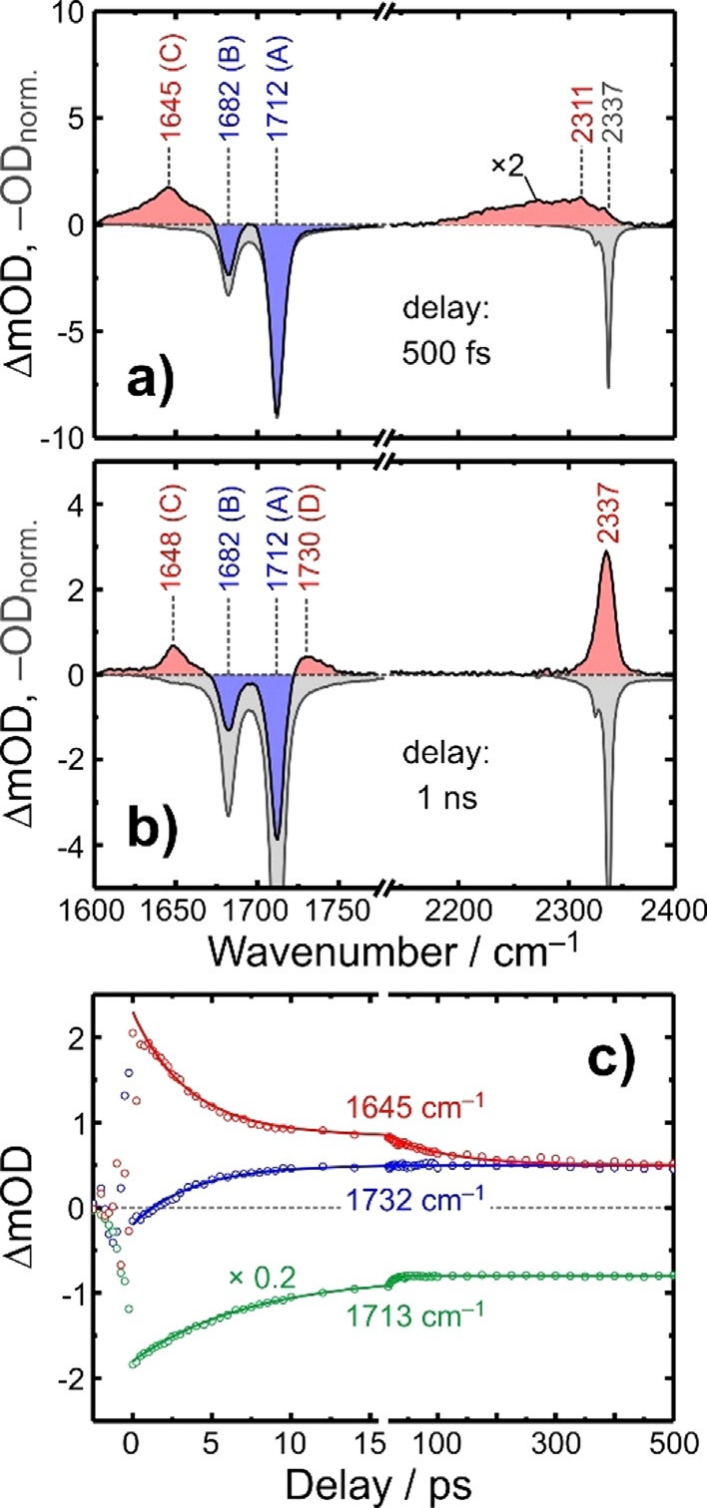
Femtosecond 266 nm‐pump/mid‐IR probe spectra of [**1**] in DMSO solution at room temperature at a very short (500 fs, panel a) and a very long (1 ns, panel b) time delay. Bleaching signals are shaded in blue, transient absorption signals are highlighted in red. The inverted FTIR spectrum (gray) is scaled to match the bleaching amplitude of band A. Note the different scaling of the ordinates in panels (a) and (b). c) Representative kinetic traces. Symbol are experimental data and solid curves are exponential fits.

A UV/MIR‐spectrum recorded at a delay of 500 fs after 266 nm‐photolysis of [**1**] in liquid DMSO solution is displayed in Figure 3 a. Focusing first on the oxalate's C=O‐stretching region, we observe three distinct pump‐induced signals. (i) An increased transmission (also called a bleach, corresponding to a negative differential optical density, ΔOD) exactly at the spectral position of band A; (ii) a similar increased transmission precisely at the position of band B; and (iii) an increased absorption (i.e. ΔOD>0) at a probe wavenumber of 1645 cm^−1^ (from hereon denoted band C). The bleaching signals, (i) and (ii), originate from the depletion of the population in the ground state of [**1**] as a result of the optical excitation. The spectral shape of the bleaching bands follows the (inverted) stationary FTIR‐spectrum of the sample. The induced absorption band C on the other hand, indicates the presence of nascent species that are absent in the sample prior to its interaction with the pump pulse and that are generated only in response to the ultraviolet excitation of [**1**].

Simultaneously, an additional, extremely broad transient absorption appears at 2311 cm^−1^, that is, in a spectral region, where the antisymmetric stretching absorption of carbon dioxide is expected to appear (cf. also Figure 2 b, right abscissa, for a stationary FTIR‐spectrum of liquid DMSO purged with gaseous CO_2_). This signal is unequivocal evidence that [**1**] is photolytically transformed by losing a neutral CO_2_ molecule within only 500 fs after 266 nm‐absorption. The enormous spectral width of this band indicates that excess vibrational energy is deposited in the triatomic fragment during the photolysis. We have recently reported and analyzed the same spectral signature of a vibrationally “hot” CO_2_‐fragment emerging from the photolysis of ferrioxalate.^[11]^ Having identified CO_2_ as a primary product we are led to conclude that the absorptive signal (C) stems from the geminate partner, which in turn, has to be a nascent Fe‐containing complex that still absorbs in the C=O‐stretching region at 1645 cm^−1^. Therefore, band C must be attributed to a species of the general formula, [Fe(cyclam)(CO_2_)]^+^; or in other words, a carbon dioxide complex of iron has formed within 500 fs according to the photoreaction

(1) $$\left[\mathrm{Fe}{}^{\mathrm{III}}\left(\mathrm{cyclam}\right)\left(C{}_{2}O{}_{4}\right)\right]{}^{+}\;+\;h\nu \;\to \;\left[\mathrm{Fe}{}^{?}\left(\mathrm{cyclam}\right)\left(\mathrm{CO}{}_{2}\right)\right]{}^{+}\;+\;\mathrm{CO}{}_{2}$$

Its molecular and electronic structure (i.e. binding mode, oxidation state, and nature of the CO_2_‐ligand) is yet to be determined. A primary quantum yield for the photochemical conversion of [**1**] of 44 % can be estimated from the magnitude of the pump‐induced CO_2_‐signal and the known extinction coefficient of the antisymmetric CO_2_‐stretching mode (Figure 2 b).^[11]^

A UV/MIR spectrum recorded at a very long time‐delay of 1 ns is reproduced in Figure 3 b. The full spectro‐temporal evolution from 500 fs all the way up to 1 ns is displayed in the SI (Figure S7 and animation A1). Firstly, the amplitudes of the negative bleaching signals have decreased significantly indicating that the ground state has partially replenished. The bleach recovery amounts to 56 %, consistent with the yield of CO_2_‐photoproduction. Thus, the primary quantum yield is limited by non‐reactive internal conversion.^[20]^ Secondly, the induced absorption band C has experienced a pronounced spectral narrowing, a very slight frequency‐upshift from 1645 cm^−1^ to 1648 cm^−1^, and an overall loss of band integral. Thirdly, another induced absorption (denoted band D), has appeared within 1 ns, which is located at the high‐frequency edge of the bleaching bands, A and B, that is, at 1730 cm^−1^. Apparently, this secondary D‐band is due to another Fe‐CO_2_ complex, which differs distinctly from the primary metal‐containing fragment in terms of its molecular, vibrational, and electronic structures.

Kinetic traces recorded on the A‐band bleach and the induced absorptions, C and D, are displayed in Figure 3 c together with exponential fits. The bleach recovers with a single time constant of 7.4 ps. In contrast, the C‐band decays biexponentially with time constants of 3.4 ps (88 % relative amplitude) and 100 ps (22 %) while the D‐band builds up monoexponentially with 3.8 ps Thus, the species absorbing at 1730 cm^−1^ (band D) seems to be formed from the primary product absorbing at 1645 cm^−1^ (band C). Finally, the broad CO_2_‐absorption originally peaking at 2311 cm^−1^ has evolved into a narrow band that spectrally coincides exactly with the stationary absorption of CO_2_ in liquid DMSO. Clearly, the carbonaceous fragment loses its excess vibrational energy and fully thermalizes in the solvent within 1 ns.

**Figure 4:**
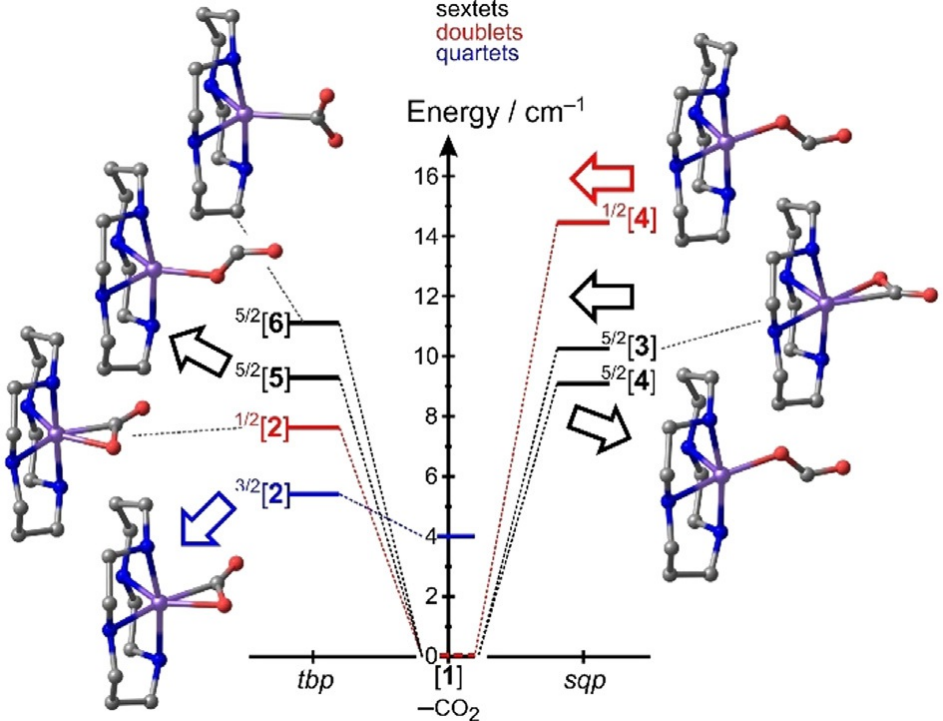
DFT‐energetics of the photolysis of [**1**] from the mPW1B95‐functional. The most stable photoproduct is species, [**2**], featuring the “side‐on” binding mode (bottom left). It has an intermediate spin ground‐state with a doublet‐quartet gap of 2213 cm^−1^. Note that the structures are grouped by the ligand sphere geometry, that is, trigonal‐bipyramidal on the left and square‐pyramidal on the right. The Cartesian coordinates of each structure are given in the Supporting Information.

To understand the origin of the induced absorptions in the C=O stretching region at early and at late delays (i.e. bands C and D), and to elucidate the underlying dynamics associated with the formation of the primary and secondary Fe‐CO_2_ complexes we resort to our DFT‐calculations (cf. SI, Table S2, S3 and Figure S8, S9). To this end, we carried out a series of geometry optimizations with a variety of different starting structures of the complex with the general formula, [Fe(cyclam)(CO_2_)]^+^. These initial guesses represented the well‐known $\eta_{C}^{1}$ and $\;\eta_{CO}^{2}$ binding modes as well as the exceptional $\eta_{O,\;bent}^{1}$ ‐motif with the bent (CO_2_^.−^)‐ligand, which we recently discovered in the solution‐phase femtochemistry of ferrioxalate.^[11]^ All three binding modes were installed in both, the axial (*ax*) and equatorial (*eq*) positions of the trigonal‐bipyramidal (*tbp*) coordination sphere, and likewise, in the apical (*ap*) and basal (*bas*) sites of the square pyramidal (*sqp*) ligand sphere. Following their in silico construction, these starting geometries were structurally optimized on the potential energy surfaces (PES) of all feasible spin multiplicities; that is, they were relaxed on each, the doublet (*S*=1/2), the quartet (*S*=3/2), and the sextet (*S*=5/2) surface. Finally, a normal mode analysis was conducted on each optimized geometry to verify that it corresponded indeed to a local or even the global minimum of the respective PES. The resultant energetics of [Fe(cyclam)(CO_2_)]^+^ relative to that of [**1**] and the molecular structures associated with stationary points on the three PESs are depicted in Figure 4. We strongly emphasize at this point that the two chosen functionals, mPW1B95 und PBE0, provide fully consistent results with regard to structure, energetics, and vibrational spectroscopy.

**Figure 5:**
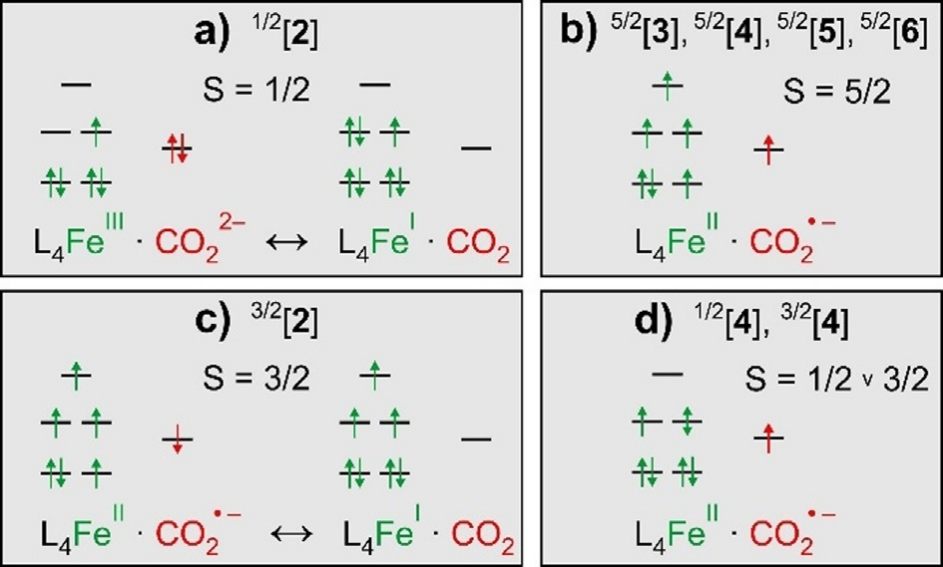
a) Limiting electronic structures of ^1/2^[**2**]: A (CO_2_^2−^)‐dianion attached to low‐spin iron(III) (*S*=1/2) and a neutral CO_2_ linked to low‐spin iron(I) (*S*=1/2). b) Electronic structure of high‐spin [Fe(cyclam)(CO_2_)]^+^: An anionic (CO_2_^.−^)‐ligand ferromagnetically coupled to high‐spin iron(II). c) Limiting electronic structures of ^3/2^[**2**]: A (CO_2_^.−^)‐radical anion (*S*=1/2) that is antiferromagnetically coupled to a high‐spin iron(II) center (*S*=2) and a neutral CO_2_ attached to a high‐spin iron(I) center (*S*=3/2). d) Electronic structure of low‐spin or intermediate‐spin Fe‐CO_2_ complexes with the bent‐O‐“end‐on” binding mode. Species with electronic structures (a) and (c) absorb frequency‐upshifted, those of the type (b) and (d) absorb frequency‐downshifted relative to the parent, [**1**].

Our in silico results can be summarized succinctly as follows: just as we expected, the “side‐on”‐motif is favored on the low‐spin (*S*=1/2) PES whereas the “Y‐on” and bent‐O‐“end‐on” motifs are preferred on the high‐spin (*S*=5/2) PES. The bonding situation aside, their electronic structures can be understood in primitive terms using the mesomeric structures given in Figure 5 a and b.

Much to our surprise however, two *electronically distinct* “side‐on” modes exist: one with the fully closed‐shell CO_2_‐ligand (*S*=1/2, Figure 5 a) and another, unexpected one with a CO_2_‐ligand having partial open‐shell character (favored on the *S*=3/2 surface, cf. Figure 5 c). The overall spin quartet is realized either by coupling the anionic (CO_2_^.−^)‐ligand (*S*=1/2) antiferromagnetically to a high‐spin iron(II) center (*S*=2) or alternatively, by attaching a neutral (CO_2_^0^)‐ligand to a high‐spin iron(I) center (*S*=3/2).

It is this unexpected quartet configuration that represents the electronic ground‐state of [Fe(cyclam)(CO_2_)]^+^. Here, the bent “side‐on”‐bound CO_2_ ligand occupies an equatorial position of a distorted trigonal‐bipyramidal ligand sphere (cf. Figure 4, bottom left). The energy of this species, *tbp*‐[Fe(cyclam)(*eq*‐$\eta_{CO}^{2}$ ‐CO_2_)]^+^ (^3/2^[**2**]), together with that of a neutral CO_2_ (*S*=0) is just 64.8 kJ mol^−1^ (or equivalently, 5416 cm^−1^) larger than that of the sextet ground‐state of [Fe(cyclam)(C_2_O_4_)]^+^. A spin‐density of half an electron residing on the triatomic ligand suggests that the electronic structure is nearly a perfect 1:1 mixture of the two limiting resonance structures given in Figure 5 c.

Guided by Figure 5, we can qualitatively predict the vibrational spectroscopy of the various structures of [Fe(cyclam)(CO_2_)]^+^. The IR‐absorption of the antisymmetric stretching vibration of the free carbon dioxide radical anion (CO_2_^.−^) in cryogenic inert matrices has been observed^[21]^ at 1657 cm^−1^; i.e. frequency‐downshifted with respect to the C=O‐resonances of the parent complex, [**1**] (cf. Figure 1). Therefore, high‐spin products (*S*=5/2) can be expected to absorb at wavenumbers below those of the two parent bands, A and B, because they all contain the (CO_2_^.−^)‐ radical anion ligand. Such products are the $\eta_{O,bent}^{1}$ ‐species, ^5/2^[**4**] and ^5/2^[**5**], and the $\eta_{C}^{1}$ ‐species, ^5/2^[**6**].

In contrast, products featuring the $\eta_{CO}^{2}$ ‐mode can be expected to absorb frequency‐upshifted relative to bands A and B because the asymmetric stretching frequency of free CO_2_ is way up at 2337 cm^−1^ and, according to Figures 5 a and c, some character of a neutral CO_2_‐ligand is mixed into their electronic and vibrational wavefunctions. Such products are the species, ^3/2^[**2**] and ^1/2^[**2**]; that is, the quartet and doublet states of the “side‐on” complex, *tbp*‐[Fe(cyclam)(*eq*‐$\eta_{CO}^{2}$ ‐CO_2_)]^+^ (see again Figure 4 and 5 a,c). Thus, we can crudely predict here that bent‐O‐“end‐on” and “Y‐on” complexes absorb red‐shifted, “side‐on” complexes absorb blue‐shifted relative to the parent.

**Figure 6:**
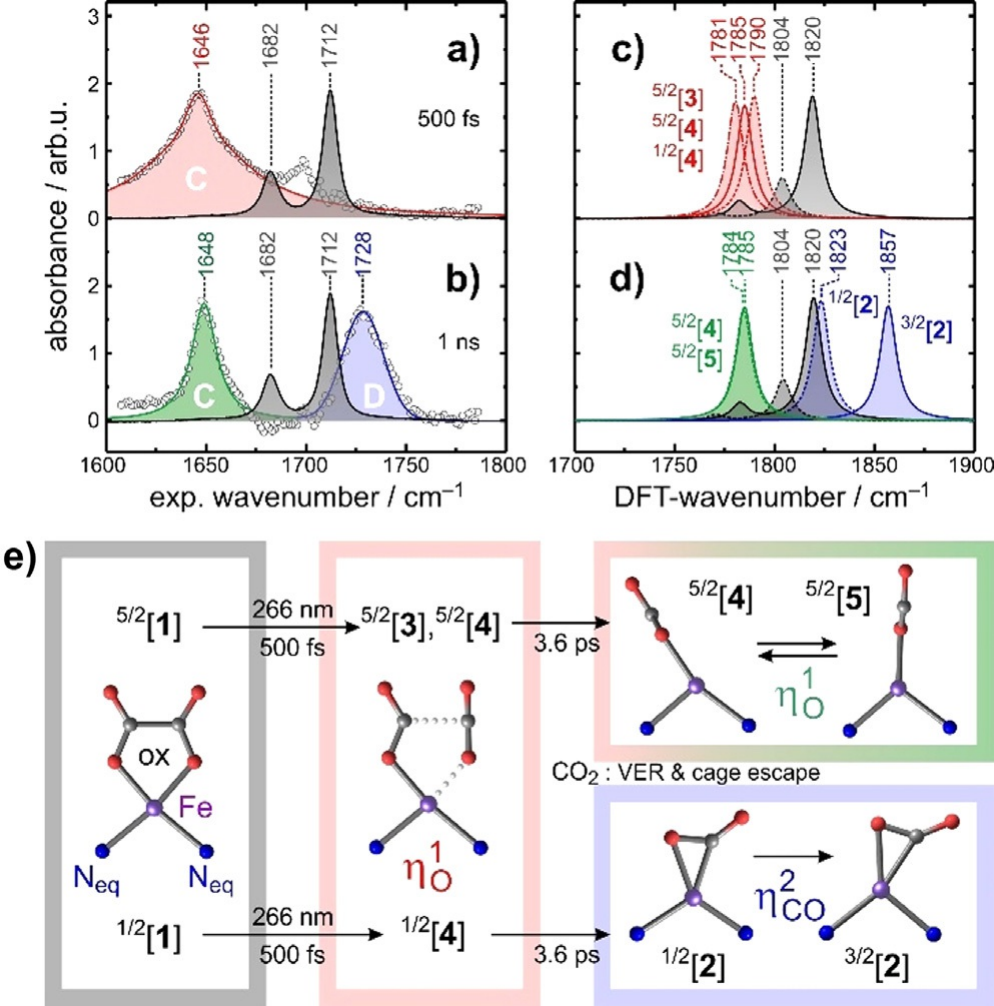
a) and b) Purely absorptive product spectra at delays of 500 fs and 1 ns exhibiting frequency‐downshifted (red and green) and upshifted (blue) bands relative to parent bands (gray). Solid curves represent fits of the data to Lorentzian and/or Gaussian line shapes. c) and d) Computational IR‐spectra for high‐spin and low‐spin complexes with the bent‐O‐“end‐on” (red and green) and the “side‐on”‐mode (blue). The parent spectra are also shown for reference purposes (solid gray is for the high‐spin and dashed gray for the low‐spin complex). e) Structural changes in the equatorial plane of the parent's ligand sphere during the photo‐induced processes following 266‐nm excitation. For clarity, C and H atoms of the cyclam ligand are hidden as are the two N‐atoms occupying the axial positions in the intact parent.

Figure 6 a and b, display so‐called purely absorptive product spectra^[22]^ at a delay of 500 fs and 1 ns, respectively. Such spectra are derived from the raw data by adding the properly weighted stationary FTIR‐spectrum of the sample thereby removing the perturbing bleaching signals from the transient absorptions of the primary and secondary Fe‐CO_2_ product complexes of interest. The spectral narrowing of the red‐shifted C‐band as well as the appearance of the blue‐shifted D‐band with increasing delay is now clearly evident. Representative DFT‐computed spectra are displayed in Figure 6 c and d and fully confirm our above qualitative notions about the spin‐state‐dependent vibrational spectroscopy of [Fe(cyclam)(CO_2_)]^+^. All complexes featuring a genuine (CO_2_^.−^)‐ligand with a spin density of one full electron absorb indeed on the low‐frequency side of the parent bands. These are the species, ^5/2^[**3**], ^5/2^[**4**], ^5/2^[**5**], and ^1/2^[**4**]. In contrast, the complexes with $\eta_{CO}^{2}$ ‐binding mode, that is, ^3/2^[**2**] and ^1/2^[**2**], absorb just as predicted on the high‐frequency side of the parent bands.

With these findings, the structural modifications during the photolysis of [**1**] are now easily derived from the femtosecond data and are summarized in Figure 6 e). The changes are best seen from above the parent's equatorial ligand plane containing all six oxalate atoms, the Fe‐center, and two of cyclam's four N‐atoms. The dynamics start from the sextet ground‐state of the parent, ^5/2^[**1**], with some thermal population in the doublet excited state, ^1/2^[**1**]. Within 500 fs, a neutral CO_2_‐molecule is excised from the C_2_O_4_‐moiety by breaking the Fe−O bond and the oxalate's C−C bond. Due to the short time scale, the CO_2_‐fragment likely remains in close proximity to the iron center. The bond cleavages generate $\eta_{O,bent}^{1}$ ‐species on both, the high‐spin and low‐spin PES and their C=O absorptions are red‐shifted relative to those of [**1**] (cf. Figure 6 a and c). The inhomogeneous distribution of primary products gives rise to the large spectral breadth of the C‐band and can be traced back to the mixed spin‐population of the parent (vide supra).

The spectral narrowing of the C‐band (cf. Figure 6 a and b) with increasing time delay demonstrates that the primary products thermalize and structurally relax to leave behind only the most stable, $\eta_{O,bent}^{1}$ ‐isomers on the sextet PES; namely, the energetically nearly degenerate species, ^5/2^[**4**] and ^5/2^[**5**]. Most importantly, however, “side‐on”‐complexes also emerge as indicated by the appearance of the characteristic blue‐shifted absorption (Figure 6 b and d). On the low‐spin surface, the $\eta_{CO}^{2}$ ‐species, ^1/2^[**2**], can be formed directly from the primary bent‐O‐“end‐on”‐species, ^1/2^[**4**], which arises through direct optical excitation of ^1/2^[**1**]. This conversion from $\eta_{O,bent}^{1}$ to $\eta_{CO}^{2}$ rationalizes the appearance of band D at the expense of band C. According to the kinetic traces in Figure 3 c, this “end‐on”‐to‐“side‐on” structural isomerization occurs with a time constant of 3.6 ps. The “side‐on”‐mode constitutes the global minimum energy structure on the low‐spin PES. It is nonetheless meta‐stable and can relax without significant structural distortions via intersystem‐crossing to the intermediate‐spin ground‐state (^3/2^[**2**]). As judged by the band integrals, about 50 % of all photo‐converted complexes feature their CO_2_‐ligand in the “side‐on”‐binding mode after 1 ns. It is important to reiterate here that this binding mode did not appear at all in the pure high‐spin model system, ferrioxalate!

## Conclusion

To conclude, our data clearly confirm the predictions we made at the outset; namely that the CO_2_‐TM binding mode can indeed be controlled by the spin. Specifically, by employing a cyclic tetradentate secondary amine, we tuned the ligand field to favor the formation of low‐spin and intermediate‐spin products in the photolysis of a photolabile oxalatoiron(III) complex. These products in turn adopt the $\eta_{CO}^{2}$ ‐“side‐on” binding motif rather than the $\eta_{O,bent}^{1}$ ‐mode, which prevails in the photolysis of the complementary pure high‐spin system, ferrioxalate.^[11]^ The results presented here clearly highlight the importance of the spin and thus, of a judicious ligand design for activating carbon dioxide with TMs. Because of their vastly different spin‐density distributions over the triatomic ligand, it is quite possible that the two binding motifs, $\eta_{O,bent}^{1}$ and $\eta_{CO}^{2}$ , display quite diverse chemical reactivities. Only the former mode contains a genuine CO_2_^.−^ radical anion with the carbon atom already detached from the metal and as such, it may readily promote a carbon‐centered follow‐up chemistry. In contrast, the fully closed‐shell “side‐on” mode with its carbonite/CO_2_ resonance structures (Figure 5 a), vanishing spin density beyond the metal, and its C‐atom tightly locked to the iron may be more vulnerable to protonation, C−O bond scission, and/or simple electron transfer.[^7b^, ^12^]

## Conflict of interest

The authors declare no conflict of interest.

## Supporting information

As a service to our authors and readers, this journal provides supporting information supplied by the authors. Such materials are peer reviewed and may be re‐organized for online delivery, but are not copy‐edited or typeset. Technical support issues arising from supporting information (other than missing files) should be addressed to the authors.

Supplementary

Supplementary
