# Supplementary material for: Spin‐Controlled Binding of Carbon Dioxide by an Iron Center: Insights from Ultrafast Mid‐Infrared Spectroscopy
Source: Angew Chem Int Ed Engl. 2020 Nov 27;60(5):2519–25. doi: 10.1002/anie.202012739 (PMC7898313; doi:10.1002/anie.202012739)
Supplement: Supplementary file 1 — Supplementary [file ANIE-60-2519-s001.pdf]

Angewandte Chemie
Eine Zeitschrift der Gesellschaft Deutscher Chemiker

Supporting Information

Spin-Controlled Binding of Carbon Dioxide by an Iron Center: Insights from Ultrafast Mid-Infrared Spectroscopy

*Steffen Straub and Peter Vöhringer**

anie_202012739_sm_miscellaneous_information.pdf
anie_202012739_sm_Movie.mp4

Table of Contents:

1. Computational
2. Experimental
3. Time-dependent density functional theory
4. Temperature-dependent FTIR-spectra
5. Magnetometry
6. EPR-spectroscopy
7. Spin-state energetics of Fe(cyclam)(C_2O_4)]$^+$ from DFT calculations
8. Infrared spectroscopy of Fe(cyclam)(C_2O_4)]$^+$ from DFT calculations
9. Full MIR-spectro-temporal evolution
10. Energetics and infrared spectroscopy of Fe(cyclam)(CO_2)]$^+$ from DFT calculations (mPW1B95)
11. Energetics and infrared spectroscopy of Fe(cyclam)(CO_2)]$^+$ from DFT calculations (PBE0)
12. Cartesian coordinates and spin densities of DFT-optimized structures
13. References

1. Computational

Electronic structure calculations were carried out within the framework of density functional theory using Gaussian 16.[1] A variety of exchange correlation functionals (cf. Table S1, columns 1-6) were used including pure GGA and pure meta-GGA functionals, hybrid-functionals with different admixtures of Hartree-Fock exchange (HFX), a long-range corrected (LC) hybrid functional, three hybrid-meta-GGA functionals and finally, three double hybrid functionals with different HFX/MP2-composition. In all calculations, a mixed Karlsruhe basis set[2] (abbreviated as def2-TZ(S)VP) was used, which included the def2-TZVP basis for the iron center, the cyclam's nitrogen atoms and the atoms of either the oxalate or the carbon dioxide ligand. The smaller def2-SVP basis was used for the remaining carbon and hydrogen atoms of the cyclam ligand. Whenever pure functionals were employed, density fitting was applied to the Coulomb problem[3] to speed up the calculation. To this end, the Weigend's auxiliary basis, W06, was assigned to all atoms.[4] For all model chemistries, the UltraFine integration grid was used except for the mPW1B95[5] and PW6B95[6] functionals, which required the SuperFine grid. Solvent effects were accounted for using the conductor-like polarizable continuum model (CPCM).[7] In some cases, an empirical dispersion correction was performed using Grimme's D3 version with Becke-Johnson damping.[8]

Geometry optimizations were carried out with Tight convergence settings and were followed by a calculation of analytical frequencies and associated dipole derivatives. The lack of imaginary frequencies verified that all optimized geometries corresponded to global or local minima on the potential energy surfaces of the parent or CO_2 product complexes. IR-spectra were then generated by convoluting the theoretical stick spectra with a Lorentzian profile having a full width at half maximum of 15 cm^{-1}. The linear absorption spectrum was predicted for both, the doublet and sextet states, of $Fe(cyclam)(ox)]^+$, using time-dependent DFT in the Tamm-Dancoff approximation.[9] To this end, the range-separated functional, ωB97XD,[10] was employed using the same mixed basis set described above. UV/Vis-spectra were then generated by convoluting the theoretical stick spectra with a Gaussian profile having a full width at half maximum of 2500 cm^{-1}. The nature of the electronic excitations to the various excited states was further explored by inspecting the natural transition orbitals.[11]

2. Experimental

UV-pump/mid-IR probe spectroscopy was conducted with a setup described in detail elsewhere.[12] Briefly, a commercial ultrafast front-end laser (Solstice Ace, Newport/Spectra Physics, output power: 6 W, repetition rate: 1 kHz, pulse duration: 50 fs, center wavelength: 800 nm) was used to drive a home-built optical parametric amplifier (OPA) with a pump energy of 350 µJ. Signal and idler pulses of the OPA were frequency-downconverted to the mid-IR region via difference-frequency generation in a type-I $AgGaS_2$-crystal thereby providing probe pulses that were fully tunable between 2.8 µm and ~10 µm and had an energy of up to 3 µJ. These probe pulse were sent through a Ge-filter to suppress unconverted signal/idler light and subsequently split into detection and reference pulses with roughly equal intensities. Pump pulses with a wavelength of 266 nm were generated by third harmonic generation of a fraction of the front-end output. To this end, two type-I-β-BaB_2O_4-crystals were employed to facilitate frequency doubling of the 800 nm-pulses in a first stage and sum-frequency generation of the resultant 400 nm pulses with the unconverted 800 nm-light in a second stage. The detection and reference pulses were focused into the sample using a Au-coated 90° off-axis parabola (10 cm effective focal length). Their beam waist inside the sample was 400 µm. Behind the sample, the mid-IR pulses were re-collimated with an identical parabola and then imaged separately onto the two

entrance slits of an imaging spectrometer (Horiba, iHR 320). A referenced detection of the pump-induced optical density was accomplished using a 2 x 32 pixel HgCdTe-array detector (Infrared associates) attached to one of the spectrometer's exit slits and a rotating chopper disk blocking every other pump pulse. Using a fused silica lens (f = 400 mm), the diameter of the pump-beam was reduced to ~1 mm at the position of the sample where it spatially overlapped with that of the detection pulses at a crossing angle of 5°. The relative pump-probe delay was adjusted with a Ag-coated hollow-roof retroreflector that was inserted into the beam path of the detection pulses and mounted on a motorized translation stage (Physik Instrumente, VT-80). The relative pump-probe polarization was set to the magic angle to remove signal contributions arising from rotational dynamics. The sample solution was circulated with a gear pump (flow rate of ca. 100 mL min^{-1}) through a home-built flow cell equipped with two CaF_2 windows whose distance was set to 100 µm with a lead spacer. The pump-probe setup was purged with N_2 to avoid atmospheric absorptions on the probe pulses.
The compound, [Fe(cyclam)(C_2O_4)][PF_6] ([**1**][PF_6]), was synthesized as follows. Upon dissolving 1.14 g of oxalic acid (12.6 mmol, 1.5 eq) in 180 mL of deionized water, 3.08 g of *cis*-[Fe(cyclam)Cl_2]Cl[13] were added and the yellow solution was stirred for 2 h at room temperature in the dark. Subsequently 18 ml of an aqueous solution of NH_4PF_6 (2.14 mol/L, 4.5 eq) are added to the now orange solution and the mixture is stored over night at 3 °C. On the next day, the precipitated product is removed by filtration, washed with cold water and diethyl ether, and subsequently dried in vacuo at room temperature for 4 h. [**1**][PF_6]is obtained as an orange-brown solid (3.88 g, 7.99 mmol, 94% yield). Anal. calcd. for $C_{12}H_{24}N_4O_4FePF_6$: 11.45% N, 29.47% C, 4.95% H; Found: 11.23% N, 29.17% C, 5.01% H.

3. Time-dependent density functional theory

A comparison between the experimental and theoretical spectrum of [**1**] is shown in Figure S1. Highest occupied (particle, HOTO) and lowest unoccupied (hole, LUTO) orbitals for the most dominant sextet roots in the near UV are displayed in Figure S2. Except for root 10, which is predominantly a d-d-resonance, these natural transition orbitals clearly emphasize the ligand-to-metal charge transfer character of the near-UV-transitions.

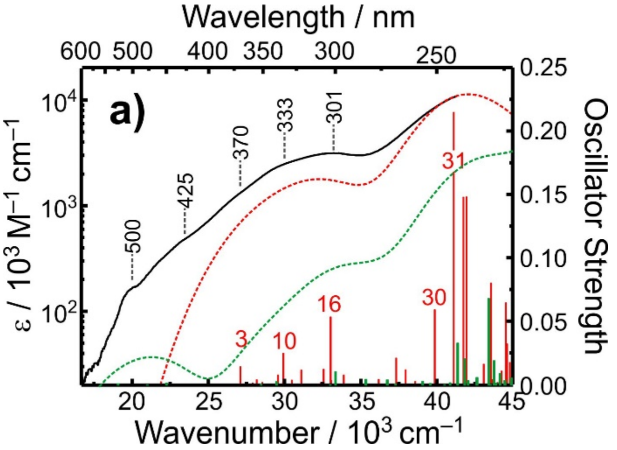

Figure S1. UV/Vis-spectrum of [**1**]. The experimental spectrum is shown in black. The dashed curves and the sticks are from the TD-DFT calculation (red: S = 5/2, green: S = 1/2). Black numbers highlight spectral positions in nm and red numbers indicate indices of the most important sextet roots.

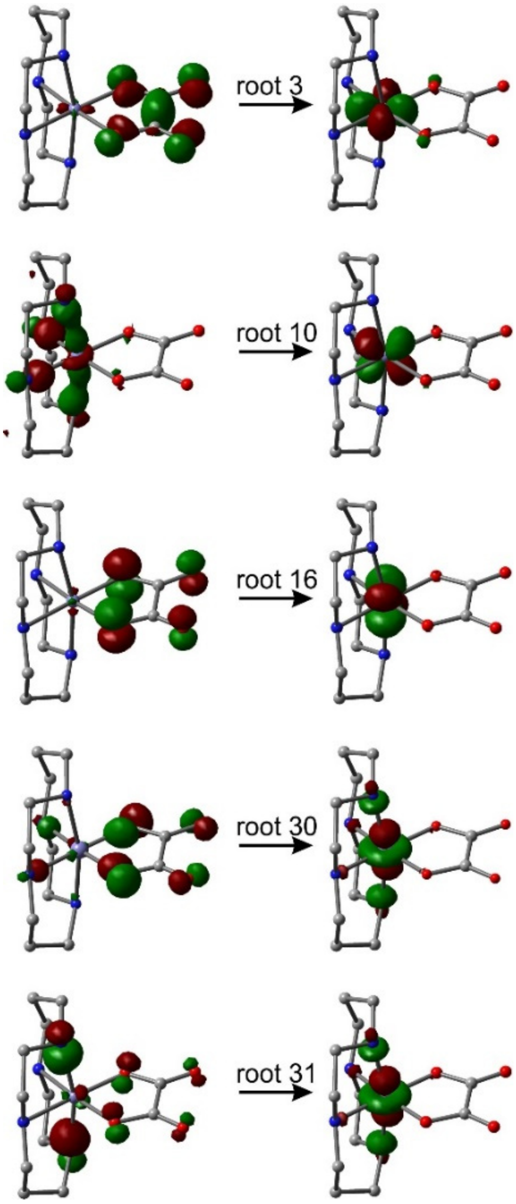

Figure S2. Surface contours (@0.06) of the natural transition orbitals of the most important sextet roots of [**1**]. HOTOs are displayed on the left and LUTOs are on the right. T

4. Temperature-dependent FTIR-spectra

The FTIR-spectra of [**1**] in liquid DMSO solution was recorded in the temperature interval between 20°C and 100°C and representative data are collected in Figure S3. It can be seen that the major bands at 1712 cm^{-1} and 1682 cm^{-1} slightly decay while at the same time, additional red-shifted absorptions appear (cf. arrows). There seem to be two isosbestic points at 1697 cm^{-1} and 1689 cm^{-1}, which is indicative of an interconversion between two states, presumably between the sextet and the doublet state. For higher temperatures, the complex eventually suffers from a chemical decay, which releases gaseous carbon dioxide. The thermal decomposition of oxalate complexes is known for quite some time and has been reported in the previous literature.[14]

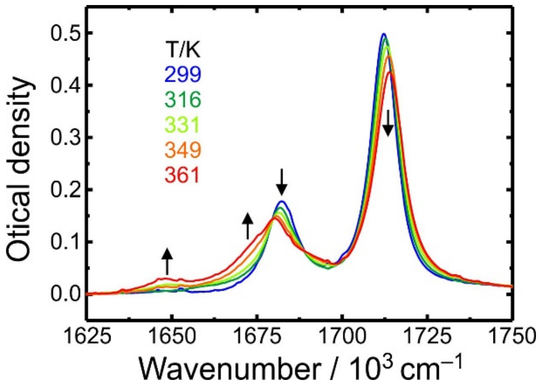

Figure S3. Representative temperature-dependent FTIR-spectra of [**1**] in liquid DMSO solution.

5. Magnetometry

Measurements of the temperature-dependent magnetic susceptibility, χ_M, were carried out using a vibrating sample magnetometer (PPMS platform from Quantum Design) in the temperature interval, 1.9 K ≤ T ≤300.0 K (cf. Figure S4) . The raw data were corrected for diamagnetic contributions arising from the sample capsule. Following the analysis described by Berry et al.[15] the product, $\chi_M T$, was fitted using a spin-crossover model assuming an ideal solution of spin carriers with S = 1/2 (Curie constant, C_{LS} = 0.375) and S = 5/2 (C_{HS} = 4.376).

$$\chi_M T = (1-f)\left[C_{HS} - (C_{LS} + \chi_{TIP}T)\left\{1 + exp\left[\frac{\Delta H}{R}\left(\frac{1}{T} - \frac{1}{T_C}\right)\right]\right\}^{-1} + (C_{LS} + \chi_{TIP}T)\right] + fC_{HS} \qquad (1)$$

Here, R is the universal gas constant, ΔH and ΔS are the enthalpy and entropy, respectively, of the spin-crossover transition, and T_C = ΔH/ΔS is the transition temperature. Moreover, a small temperature-independent paramagnetism, χ_{TIP}, of 5 × 10^{-3} cm^3 / mol was added for the doublet state. Finally, the fit accounts for a fraction, f, of 8.5% of a paramagnetic species with S = 5/2 that persists even at low temperatures. From least-squares fitting, we obtain ΔH = 6.2 kJ/mol, T_C = 213 K, and thus, ΔS = 29 J/(mol K).

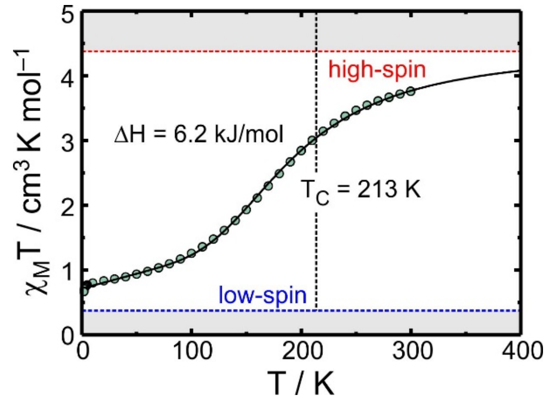

Figure S4. Temperature dependence of the magnetic susceptibility in a $\chi_M T(T)$-representation. Symbols are the experimental data and the solid curve is from equation (1). The fit parameters in given in figure and in the text. The blue and red dashed horizontal lines refer to the expected values for $\chi_M T$ of a pure doublet and sextet sample, respectively.

6. EPR-spectroscopy

The X-band EPR spectrum of a frozen solution of [Fe(cyclam)(C_2O_4)]PF_6 is displayed in Figure S5.

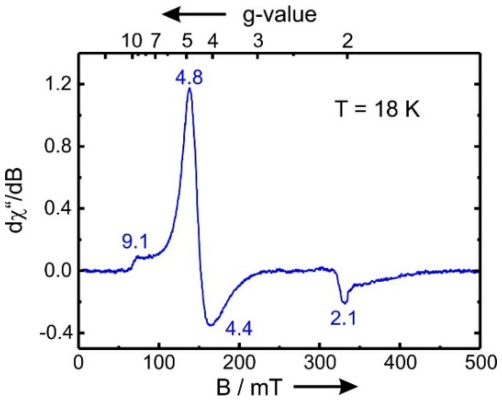

Figure S5. X-band EPR-spectrum of complex, [**1**], in frozen DMSO at 18 K. Numbers indicate g-value peak positions.

The spectrum displays two features at g ≈ 4.6 and g ≈ 9.1 in addition to a minor peak at g = 2.1, which is due to a cavity artefact. The data shown in Figure S4 are remarkably similar to the complex, *cis*-[Fe(cyclam)(N_3)$_2$]$^+$, which was successfully modeled in terms of a high-spin (S = 5/2) iron(III) species.[31] Thus, we can conclude that in DMSO solution at 18 K, the complex occupies a high-spin ground state.

7. Spin-state energetics of Fe(cyclam)(C_2O_4)]$^+$ from DFT calculations

Table S1. Relative energy, ${}^{S}E_{rel}$, in wavenumbers of the doublet (S = 1/2), quartet (S = 3/2), and sextet (S = 5/2) states of [Fe(cyclam)(ox)]$^+$. The energy of the ground state is 0 cm^{-1}.

XC-fxnl.	Disp.	type	% HFX	% MP2	Ref.	${}^{1/2}E_{rel}$ in cm^{-1}	${}^{3/2}E_{rel}$ in cm^{-1}	${}^{5/2}E_{rel}$ in cm^{-1}
BP86	D3BJ	GGA			[16]	0	6599	7070
B97D3	D3BJ	GGA			[8, 17]	1210	4622	0
OPBE	D3BJ	GGA			[18]	0	4731	568
M06L		meta-GGA			[19]	1302	4887	0
TPSS	D3BJ	meta-GGA			[20]	0	7790	7686
B3(V5)LYP*	D3BJ	hybrid	15		[21]	0	5043	2260
B3LYP		hybrid	20		[22]	0	3849	1039
B1LYP		hybrid	25		[23]	728	3883	0
PBE0	D3BJ	hybrid	25		[24]	1067	3899	0
PBEh1PBE		hybrid	25		[25]	504	3769	0
BH&HLYP		hybrid	50		[26]	7339	8406	0
ωB97XD	D2	LC-hybrid	19.57		[10]	0	4324	1336
TPSSh	D3BJ	hybrid meta	10		[27]	0	6049	4766
PW6B95	D3BJ	hybrid meta	28		[6]	0	4276	945
mPW1B95	D3BJ	hybrid meta	31		[5]	49	4009	0
PBE0DH		double hybrid	50	12.5	[28]	9334		0
B2PLYP	D3BJ	double hybrid	53	27	[29]	874	8166	0
DSDPBEP86		double hybrid	72	36/51	[30]	17859		0

8. Infrared spectroscopy of $Fe(cyclam)(C_2O_4)]^+$ from DFT calculations

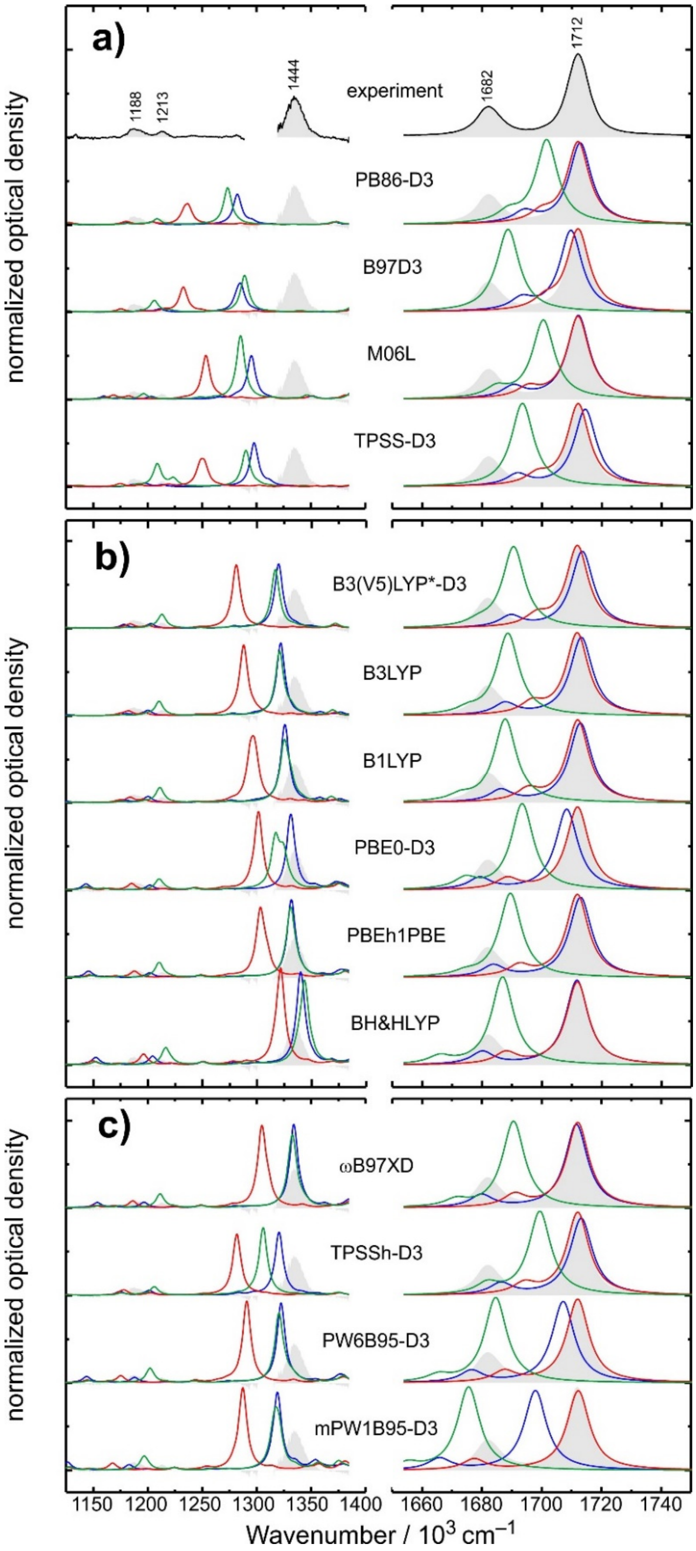

Figure S6. Comparison of the experimental FTIR-spectrum of $Fe(cyclam)(ox)]^+$ in DMSO ((gray shaded spectrum) with computational spectra obtained from various DFT-methods for the doublet (blue), quartet (green) and sextet (red) states. a) pure GGA and meta-GGA exchange correlation functionals, b) hybrid GGA-funcationals with increasing admixture of exact exchange (from top to bottom) and c) long-range-corrected hybrid and hybrid-meta-GGA functionals. For each method, the predicted vibrational frequencies were scaled such that the asymmetric CO-stretching band of the sextet state lines up with its experimental counterpart. The def2-TZ(S)VP basis set was used throughout.

9. Full MIR-spectro-temporal evolution

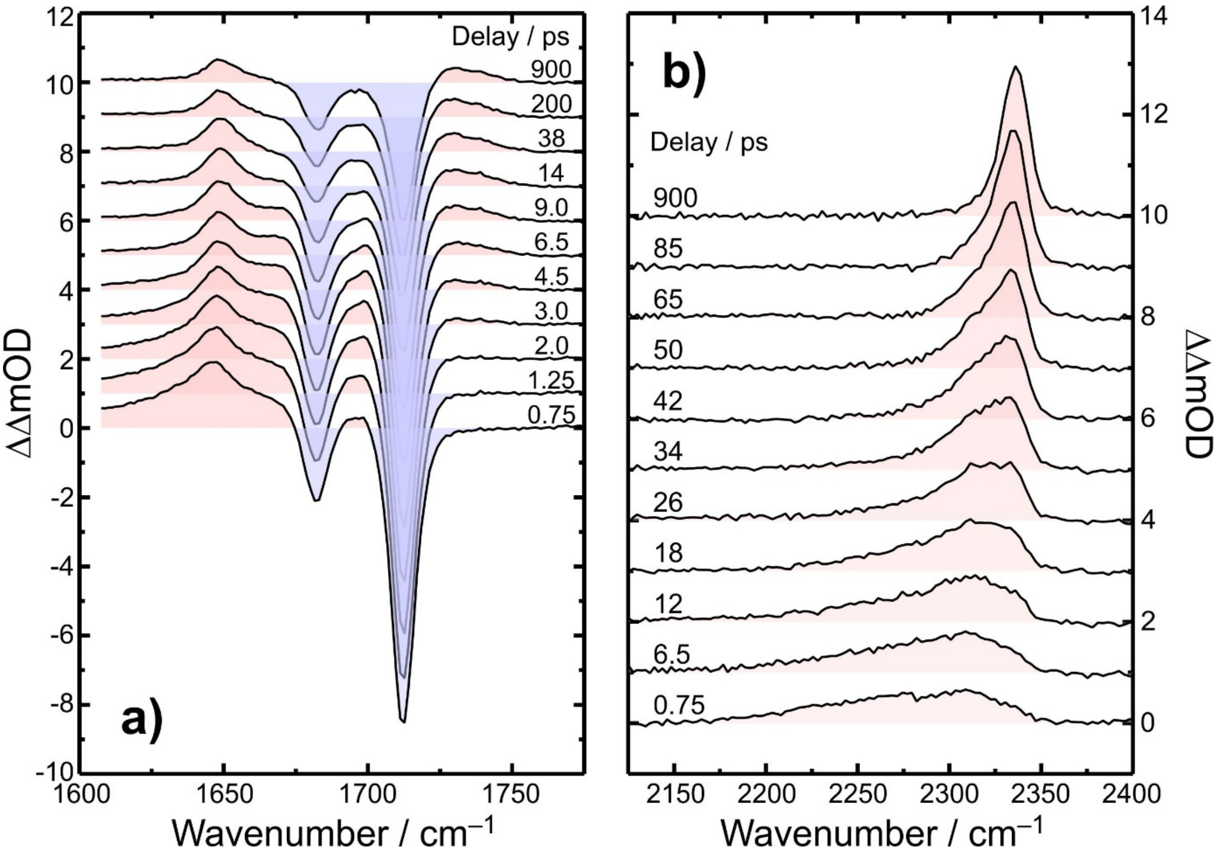

Figure S7. Representative UV-pump/MIR-probe spectra recorded in the C=O stretching region (left panel) and in the CO_2-stretching region right panel. Spectra are shifted vertically by a successive increment of 1 ΔmOD.

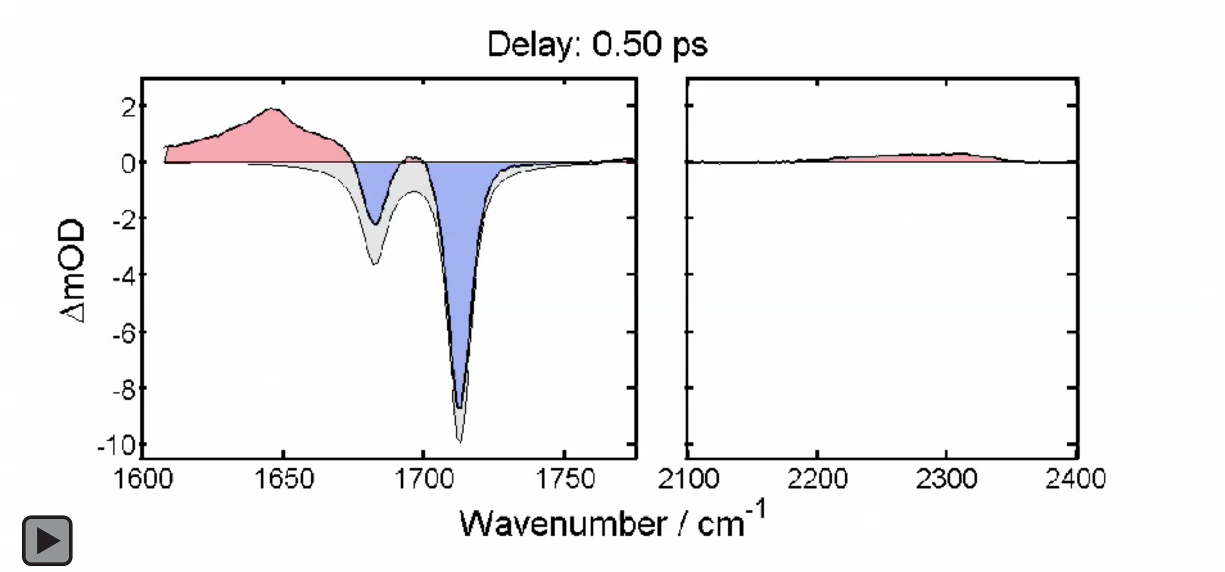

Animation A1. Full MIR-spectro-temporal evolution in the C=O and CO_2-stretching spectral region (click on the arrow to start the animation or view the separate MP4-file in an external movie player)

10. Energetics and Infrared spectroscopy of $Fe(cyclam)(CO_2)]^+$ from DFT calculations (mPW1B95).

Table S2. Relative energies, E_{rel}, and unscaled (harmonic) asymmetric CO_2-stretching frequencies, $\tilde{\nu}$, of the "side-on" (η^2_{CO}), "O-end-on" (η^1_O), and "Y-on" (η^1_C) complexes of $[Fe(cyclam)(CO_2)]^+$ in the doublet (S = 1/2), quartet (S = 3/2), and sextet (S = 5/2) states. Level of theory: mPW1B95-D3(BJ)/def2-TZ(S)VP with CPCM(DMSO) solvation. Energies are referenced to that of the parent in the ground-state state from which the energy of the neutral CO_2-fragment was subtracted. Therefore, they can be regarded as reaction energies at T = 0 K for the transformation, $[Fe(cyclam)(ox)]^+$ (S = 5/2) $\rightarrow$ $[Fe(cyclam)(CO_2)]^+$ (S) + CO_2 (S = 0).

binding mode	S	trigonal-bipyramidal (tbpy)			square-pyramidal (sqpy)		
		E_{rel} / cm^{-1}	$\tilde{\nu}$ / cm^{-1}	coord. site	E_{rel} / cm^{-1}	$\tilde{\nu}$ / cm^{-1}	coord. site
η^2_{CO}	1/2	**7629**	**1823.41**	**equat.**			
	3/2	**5416**	**1857.17**	**equat.**			
	5/2				10252	1790.19	basal
$\eta^1_{O,bent}$	1/2				14448	1780.70	basal
	3/2				12645	1781.64	basal
	5/2	9286	1784.33	equat.	**9090**	**1785.36**	**basal**
η^1_C	5/2	11106	1786.04	equat.			

(color coding of table entries: green = species [**2**], yellow = [**3**], orange = [**4**], blue = [**5**], green = [**6**])

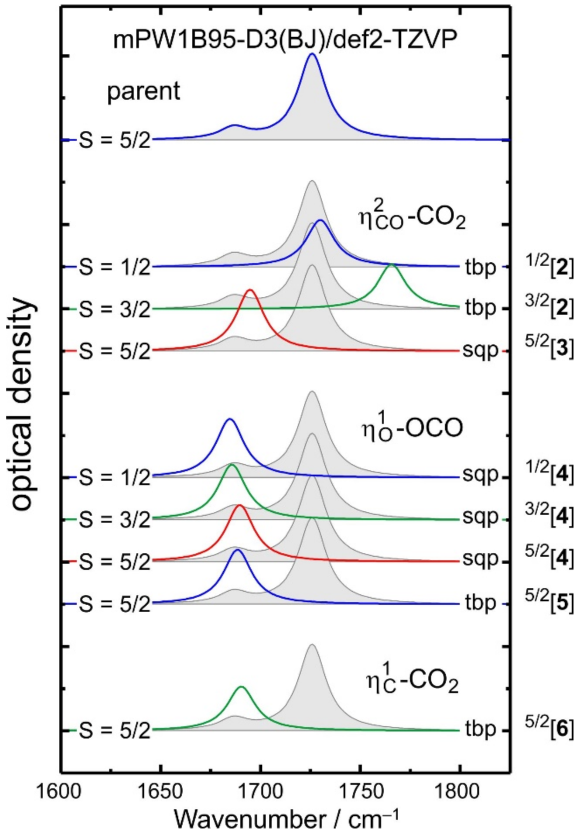

Figure S8. Computational IR-spectra of the ground-state parent complex and the various isomers of $[Fe(cyclam)(CO_2)]^+$ in the doublet (S = 1/2, blue), quartet (S = 3/2, green), and sextet (S = 5/2, red) states at the level of theory, mPW1B95-D3(BJ)/def2-TZ(S)VP; CPCM(DMSO). tbp = trigonal bipyramidal, sqp = square-pyramidal. Harmonic frequencies scaled by 0.9408.

11. Energetics and Infrared spectroscopy of $[Fe(cyclam)(CO_2)]^+$ from DFT calculations (PBE0).

Table S3. Relative energies, E_{rel}, and unscaled (harmonic) asymmetric CO_2-stretching frequencies, $\tilde{\nu}$, of the "side-on" (η^2_{CO}), "O-end-on" (η^1_O), and "Y-on" (η^1_C) complexes of $[Fe(cyclam)(CO_2)]^+$ in the doublet (S = 1/2), quartet (S = 3/2), and sextet (S = 5/2) states. Level of theory: PBE0-D3(BJ)/def2TZVP with CPCM(DMSO) solvation. Energies are referenced to that of the parent in the ground-state state from which the energy of the neutral CO_2-fragment was subtracted. Therefore, they can be regarded as reaction energies at T = 0 K for the transformation, $[Fe(cyclam)(ox)]^+$ (S = 5/2) $\rightarrow$ $[Fe(cyclam)(CO_2)]^+$ (S) + CO_2 (S = 0).

binding mode	S	trigonal-bipyramidal (tbpy)			square-pyramidal (sqpy)		
		E_{rel} / cm^{-1}	$\tilde{\nu}$ / cm^{-1}	coord. site	E_{rel} / cm^{-1}	$\tilde{\nu}$ / cm^{-1}	coord. site
η^2_{CO}	1/2	8768	1803.88	equat.			
	3/2	6062	1840.31	equat.			
	5/2				11059	1769.74	basal
$\eta^1_{O,bent}$	1/2				17137	1777.05	basal
	3/2				14013	1773.78	basal
	5/2	9862	1775.98	equat.	11267	1776.94	basal
η^1_C	5/2	11267	1764.47	equat.			

(color coding of table entries: green = species [**2**], yellow = [**3**], orange = [**4**], blue = [**5**], green = [**6**])

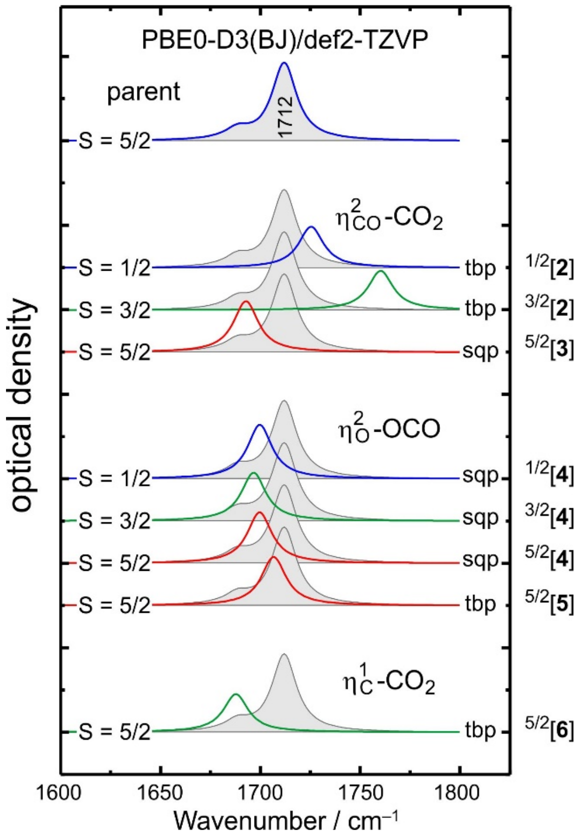

Figure S9. Computational IR-spectra of the ground-state parent complex and the various isomers of $[Fe(cyclam)(CO_2)]^+$ in the doublet (S = 1/2, blue), quartet (S = 3/2, green), and sextet (S = 5/2, red) states at the level of theory, PBE0-D3(BJ)/def2-TZ(S)VP; CPCM(DMSO). tbp = trigonal bipyramidal, sqp = square-pyramidal. Harmonic frequencies scaled by 0.9565

12. Cartesian coordinates and spin densities of DFT-optimized structures

[Fe(cyclam)(ox)]$^{+}$, S = 5/2, E = -2255.10808860

C	-3.42822	2.54168	-0.98402
C	-1.94800	2.52129	-0.50185
O	-4.01746	3.57606	-1.16197
O	-3.91225	1.35174	-1.15946
O	-1.48685	1.31797	-0.35801
O	-1.33941	3.53942	-0.29717
Fe	-2.71292	-0.14424	-0.78124
N	-1.22372	-1.56841	-0.05110
N	-2.00304	-0.38370	-2.81944
N	-4.23323	-1.51143	-1.55638
N	-3.42899	-0.43436	1.24797
H	-1.52728	-2.51507	-0.25981
H	-2.26399	0.52116	-3.19916
H	-3.94985	-2.47117	-1.38119
H	-3.14745	0.45136	1.65702
C	-0.54522	-0.52248	-2.98980
H	-0.10155	0.44048	-2.71359
H	-0.32575	-0.69396	-4.05217
C	0.08048	-1.62236	-2.15642
H	1.12024	-1.71832	-2.48993
H	-0.38331	-2.59497	-2.37428
C	0.10208	-1.38503	-0.66072
H	0.42954	-0.36366	-0.43355
H	0.81278	-2.07492	-0.18628
C	-1.22182	-1.41178	1.40967
H	-0.59755	-2.17616	1.88872
H	-0.78591	-0.43063	1.63544
C	-2.64275	-1.48569	1.90918
H	-2.68114	-1.37505	2.99947
H	-3.08309	-2.45777	1.66207
C	-4.88977	-0.54458	1.41305
H	-5.11465	-0.74237	2.46970
H	-5.31061	0.43620	1.16488
C	-5.53938	-1.60453	0.54679
H	-5.09793	-2.59368	0.73453
H	-6.58121	-1.68721	0.87740
C	-5.55524	-1.32096	-0.94078
H	-5.86219	-0.28680	-1.13610
H	-6.27920	-1.98169	-1.43626
C	-4.23226	-1.30455	-3.01090
H	-4.87399	-2.03772	-3.51503
H	-4.64601	-0.30663	-3.20208
C	-2.81382	-1.39333	-3.51509
H	-2.77428	-1.24539	-4.60094
H	-2.39540	-2.38326	-3.30355

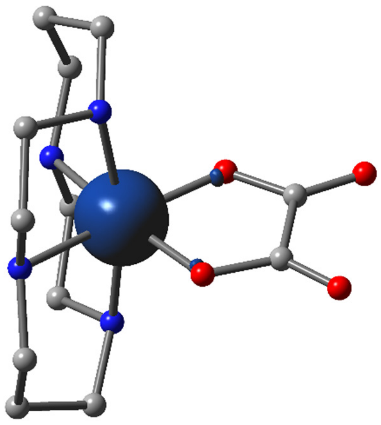

Figure S10. Spin density of $^{5/2}$[**1**].

[Fe(cyclam)(ox)]$^{+}$, S = 1/2, E = -2255.10786658

C	-3.40734	2.30620	-1.02950
C	-1.97248	2.28248	-0.45347
O	-4.00567	3.33718	-1.21219
O	-3.85779	1.12193	-1.28814
O	-1.54453	1.08152	-0.23715
O	-1.35514	3.29507	-0.23450
Fe	-2.71580	-0.29698	-0.78510
N	-1.33226	-1.63408	-0.18828
N	-2.06935	-0.31521	-2.68335
N	-4.12711	-1.58513	-1.42394
N	-3.36296	-0.36189	1.11205
H	-1.60527	-2.57359	-0.46029
H	-2.46027	0.54845	-3.04580
H	-3.87514	-2.53889	-1.18238
H	-2.95408	0.48155	1.50122
C	-0.61285	-0.27888	-2.89861
H	-0.25037	0.67608	-2.50510
H	-0.41564	-0.29358	-3.97818
C	0.11096	-1.42007	-2.22248
H	1.17024	-1.34142	-2.49272
H	-0.21497	-2.39290	-2.61316
C	0.02891	-1.39655	-0.71731
H	0.34517	-0.42021	-0.33700
H	0.69679	-2.15581	-0.29192
C	-1.30827	-1.57153	1.28525
H	-0.76759	-2.42702	1.70475
H	-0.76928	-0.65700	1.55642
C	-2.72158	-1.50599	1.77641
H	-2.76614	-1.40136	2.86682
H	-3.26194	-2.41913	1.50902
C	-4.81841	-0.30148	1.32895
H	-5.01617	-0.34751	2.40754
H	-5.16017	0.67347	0.96732
C	-5.56634	-1.40412	0.61611
H	-5.26148	-2.39570	0.97507
H	-6.62383	-1.31156	0.88900
C	-5.48306	-1.33423	-0.88754
H	-5.77719	-0.33941	-1.23645
H	-6.16746	-2.06442	-1.33692
C	-4.14877	-1.47596	-2.89477
H	-4.70664	-2.30668	-3.34073
H	-4.66837	-0.54238	-3.13737
C	-2.73379	-1.42470	-3.38271
H	-2.68497	-1.28853	-4.46947
H	-2.21322	-2.35656	-3.14178

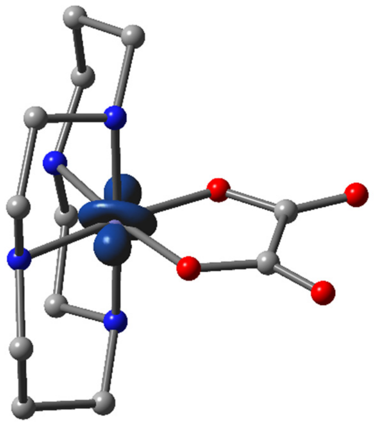

Figure S11. Spin density of $^{1/2}$[**1**].

[Fe(cyclam)(ox)]$^+$, S = 3/2, E = -2255.08982075

C	-3.42137	2.39435	-1.01529
C	-1.95954	2.37304	-0.47548
O	-4.00051	3.44099	-1.19957
O	-3.89838	1.21935	-1.22671
O	-1.50466	1.18369	-0.29680
O	-1.36104	3.40319	-0.26235
Fe	-2.71505	-0.33184	-0.78468
N	-1.21255	-1.64336	-0.07396
N	-2.05649	-0.36795	-2.66290
N	-4.24423	-1.58751	-1.53527
N	-3.37525	-0.41499	1.09159
H	-1.49146	-2.60267	-0.26180
H	-2.39003	0.54874	-2.95137
H	-3.98672	-2.55884	-1.38237
H	-3.01996	0.48297	1.41092
C	-0.59733	-0.40278	-2.87078
H	-0.19915	0.54835	-2.50385
H	-0.41153	-0.45138	-3.95124
C	0.09410	-1.55107	-2.17064
H	1.13194	-1.56439	-2.52204
H	-0.33068	-2.51732	-2.47541
C	0.11610	-1.43695	-0.66435
H	0.44879	-0.43755	-0.36324
H	0.81670	-2.16799	-0.23993
C	-1.21922	-1.44559	1.38194
H	-0.62895	-2.21428	1.89474
H	-0.75837	-0.47098	1.57908
C	-2.64722	-1.45359	1.84585
H	-2.72461	-1.25828	2.92148
H	-3.11632	-2.42252	1.64740
C	-4.83477	-0.42249	1.29874
H	-5.02245	-0.49985	2.37717
H	-5.21060	0.54836	0.96122
C	-5.55124	-1.53301	0.56404
H	-5.14924	-2.51733	0.84029
H	-6.58965	-1.53311	0.91407
C	-5.56884	-1.37301	-0.93802
H	-5.88111	-0.35841	-1.20846
H	-6.28346	-2.07656	-1.38487
C	-4.23570	-1.34002	-2.98397
H	-4.84294	-2.07852	-3.52083
H	-4.67654	-0.34991	-3.14606
C	-2.80949	-1.36159	-3.45284
H	-2.73151	-1.12644	-4.52042
H	-2.36107	-2.34743	-3.29541

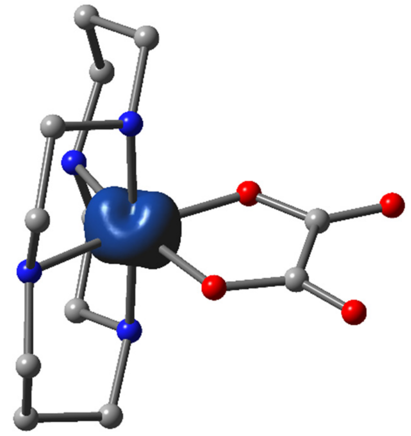

Figure S11. Spin density of [3/2][**1**].

trig.-bipyram.-[Fe(cyclam)(equat.-η^2_{CO}-CO_2)]$^+$, [1/2][**2**]; S = 1/2, E = -2066.46373336

C	-2.19590	1.34588	-0.57761
O	-1.31678	2.09508	-0.23279
O	-3.36828	1.43968	-1.02648
Fe	-2.72716	-0.43255	-0.77939
N	-1.21488	-1.54537	-0.08223
N	-2.10431	-0.40917	-2.69424
N	-4.32439	-1.58496	-1.54518
N	-3.40744	-0.45055	1.12884
H	-1.42322	-2.51179	-0.32061
H	-2.47338	0.48308	-3.00922
H	-4.12118	-2.56040	-1.35076
H	-3.07450	0.42288	1.52202
C	-0.65482	-0.38844	-2.94050
H	-0.29675	0.60681	-2.65222
H	-0.46273	-0.50927	-4.01543
C	0.10940	-1.43449	-2.15879
H	1.15133	-1.40213	-2.49779
H	-0.24692	-2.44582	-2.40224
C	0.09964	-1.23304	-0.65986
H	0.32852	-0.19031	-0.40718
H	0.86213	-1.86897	-0.18976
C	-1.24526	-1.44042	1.38444
H	-0.63341	-2.22003	1.85468
H	-0.81846	-0.46624	1.65271
C	-2.68297	-1.51993	1.82465
H	-2.77424	-1.42831	2.91449
H	-3.11421	-2.48511	1.53496
C	-4.86486	-0.45359	1.33432
H	-5.07615	-0.57743	2.40548
H	-5.22191	0.54115	1.04304
C	-5.61698	-1.50144	0.54157
H	-5.24463	-2.51092	0.76794
H	-6.65435	-1.48765	0.89589
C	-5.62884	-1.28161	-0.95561
H	-5.85955	-0.23292	-1.18386
H	-6.40943	-1.89896	-1.42302
C	-4.27248	-1.40089	-2.99802
H	-4.87426	-2.14981	-3.52942
H	-4.69605	-0.41408	-3.22320
C	-2.83094	-1.45160	-3.43171
H	-2.73581	-1.31047	-4.51621
H	-2.39461	-2.42666	-3.18544

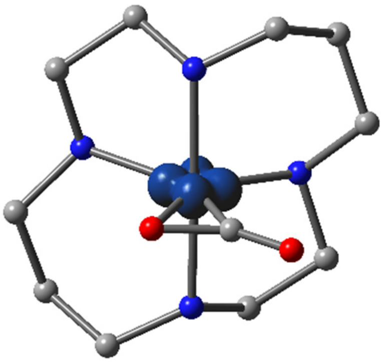

Figure S12. Spin density of [1/2][**2**].

Atomic charges and spin densities from Natural Population Analysis:

1	C	0.121324	-0.118772
2	O	-0.411392	-0.035334
3	O	-0.345866	-0.029796
4	Fe	0.209707	1.218773

Spin density on the CO_2-ligand: –0.183902

trig.-bipyram.-[Fe(cyclam)(equat.-η^2_{CO}-CO_2)]$^+$, $^{3/2}$[**2**]; S = 3/2,E = -2066.47381790

C	-1.85664	1.54735	-0.42373
O	-0.84755	2.05237	-0.02014
O	-2.98039	1.88180	-0.85184
Fe	-2.81137	-0.16049	-0.81724
N	-1.28355	-1.47488	-0.08028
N	-2.08245	-0.35618	-2.86869
N	-4.28695	-1.56016	-1.56454
N	-3.55001	-0.38039	1.24970
H	-1.54062	-2.43999	-0.26351
H	-2.37981	0.52406	-3.27424
H	-3.97821	-2.50525	-1.36149
H	-3.32678	0.50872	1.67972
C	-0.62889	-0.45693	-3.04850
H	-0.20610	0.52513	-2.80378
H	-0.39313	-0.65717	-4.10454
C	0.03287	-1.51033	-2.18072
H	1.07869	-1.57737	-2.50382
H	-0.39314	-2.50462	-2.37895
C	0.03180	-1.24342	-0.68889
H	0.30994	-0.20265	-0.48210
H	0.77885	-1.88696	-0.20164
C	-1.29261	-1.28196	1.37247
H	-0.64023	-2.00773	1.87749
H	-0.89541	-0.27866	1.57338
C	-2.70877	-1.39933	1.88298
H	-2.73114	-1.31826	2.97813
H	-3.10975	-2.38731	1.62709
C	-4.99248	-0.59589	1.42222
H	-5.21152	-0.83630	2.47355
H	-5.48680	0.35779	1.20248
C	-5.58466	-1.67544	0.53523
H	-5.09195	-2.64252	0.71315
H	-6.62124	-1.81844	0.86333
C	-5.60813	-1.38881	-0.95380
H	-5.93480	-0.35741	-1.14176
H	-6.33681	-2.05113	-1.44410
C	-4.27683	-1.39400	-3.02031
H	-4.87531	-2.16665	-3.52261
H	-4.73585	-0.42379	-3.25038
C	-2.85072	-1.42407	-3.51571
H	-2.82045	-1.33783	-4.61041
H	-2.39210	-2.38567	-3.25695

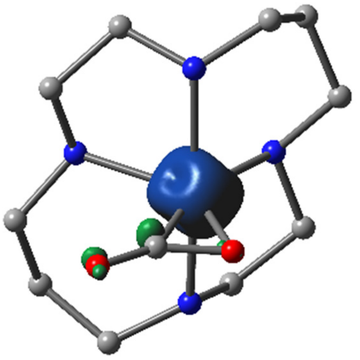

Figure S13. Spin density of $^{3/2}$[**2**].

Atomic charges and spin densities from Natural Population Analysis:

1	C	0.213187	-0.331862
2	O	-0.363431	-0.130685
3	O	-0.372026	-0.038376
4	Fe	0.480909	3.375496

Spin density on the CO_2-ligand: –0.500923

square-pyram.-[Fe(cyclam)(basal-η^2_{CO}-CO_2)]$^+$, $^{5/2}$[**3**]; S = 5/2, E = -2066.45178223

C	-3.10063	2.07454	-0.82906
O	-2.79025	3.20237	-0.53423
O	-4.05670	1.52706	-1.41301
Fe	-2.63476	-0.11733	-0.73469
N	-1.19938	-1.66176	-0.11628
N	-1.92020	-0.35123	-2.77559
N	-4.20316	-1.45382	-1.46853
N	-3.34369	-0.45513	1.28886
H	-1.54293	-2.56577	-0.42330
H	-2.19608	0.53342	-3.18857
H	-3.95216	-2.41528	-1.26298
H	-2.98557	0.35103	1.79033
C	-0.47168	-0.50354	-2.97745
H	-0.00751	0.44639	-2.68721
H	-0.26215	-0.65402	-4.04640
C	0.15842	-1.63269	-2.18465
H	1.20605	-1.69484	-2.50251
H	-0.28187	-2.60089	-2.46308
C	0.14455	-1.48382	-0.67723
H	0.49829	-0.48572	-0.38660
H	0.83581	-2.21399	-0.23172
C	-1.22711	-1.64545	1.34845
H	-0.68537	-2.50103	1.77395
H	-0.71512	-0.73407	1.68274
C	-2.66130	-1.64026	1.81997
H	-2.71002	-1.67293	2.91629
H	-3.17350	-2.53411	1.44677
C	-4.80649	-0.44918	1.46026
H	-5.05133	-0.62534	2.51755
H	-5.14764	0.56298	1.21024
C	-5.55155	-1.45385	0.60260
H	-5.21636	-2.47867	0.81706
H	-6.59912	-1.42442	0.92572
C	-5.52700	-1.20660	-0.89130
H	-5.79053	-0.16589	-1.11147
H	-6.27455	-1.84877	-1.38020
C	-4.16355	-1.28507	-2.92229
H	-4.79807	-2.02246	-3.43321
H	-4.55866	-0.28730	-3.14839
C	-2.73547	-1.40114	-3.39781
H	-2.68456	-1.34677	-4.49334
H	-2.32810	-2.37544	-3.10342

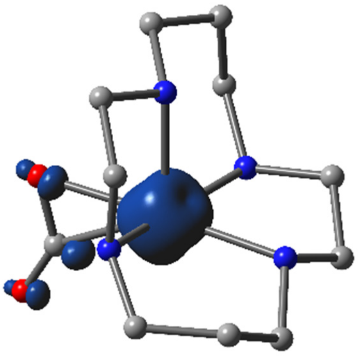

Figure S14. Spin density of $^{5/2}$[**3**].

Atomic charges and spin densities from Natural Population Analysis:

1	C	0.100410	0.471081
2	O	-0.388825	0.116025
3	O	-0.345782	0.179859
4	Fe	0.587534	4.040244

Spin density on the CO_2-ligand: 0.766965

square-pyram.-[Fe(cyclam)(basal-η_O^1-OCO)]$^+$, $^{1/2}$[**4**]; S = 1/2, E = -2066.43266712

C	-1.68294	2.03936	0.49015
O	-1.12410	3.06624	0.77427
O	-1.64854	1.29805	-0.52005
Fe	-2.76472	-0.30927	-0.84090
N	-1.28189	-1.57638	-0.18634
N	-2.14414	-0.34432	-2.76576
N	-4.19817	-1.62167	-1.46158
N	-3.40791	-0.34434	1.07962
H	-1.51657	-2.53763	-0.41162
H	-2.52939	0.49959	-3.17826
H	-3.93601	-2.56528	-1.19518
H	-3.02938	0.53409	1.43622
C	-0.68991	-0.29305	-2.98233
H	-0.35177	0.68548	-2.62515
H	-0.47893	-0.34903	-4.05971
C	0.07355	-1.38321	-2.25960
H	1.12101	-1.30523	-2.57505
H	-0.25034	-2.38108	-2.58583
C	0.04372	-1.29285	-0.75185
H	0.31569	-0.27984	-0.43435
H	0.78187	-1.98458	-0.32144
C	-1.28043	-1.44147	1.27494
H	-0.69255	-2.23303	1.75761
H	-0.80578	-0.48056	1.51131
C	-2.70388	-1.43664	1.75844
H	-2.75295	-1.33006	2.85038
H	-3.18888	-2.38513	1.50018
C	-4.85417	-0.34054	1.32138
H	-5.05106	-0.41025	2.40075
H	-5.23641	0.63005	0.98252
C	-5.58655	-1.45030	0.59743
H	-5.23426	-2.43741	0.92721
H	-6.63951	-1.40335	0.89979
C	-5.53718	-1.35934	-0.90951
H	-5.82950	-0.35250	-1.23510
H	-6.25731	-2.06229	-1.35076
C	-4.21075	-1.55768	-2.93197
H	-4.74695	-2.40927	-3.36909
H	-4.75037	-0.64588	-3.21636
C	-2.79184	-1.48764	-3.42009
H	-2.74861	-1.40811	-4.51415
H	-2.25689	-2.40007	-3.13571

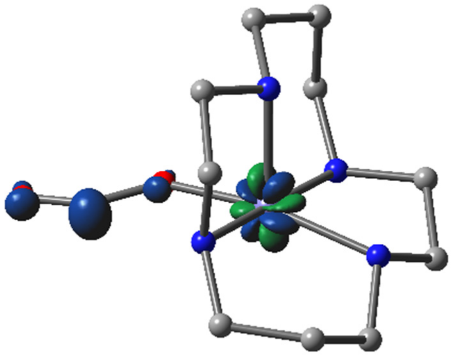

Figure S15. Spin density of $^{1/2}$[**4**].

Atomic charges and spin densities from Natural Population Analysis:

1 C		0.121629	0.677047
2 O		-0.363080	0.153408
3 O		-0.392501	0.080907
4 Fe		0.393213	0.040067

Spin density on the CO_2-ligand: 0.911362

square-pyram.-[Fe(cyclam)(basal-η_O^1-OCO)]$^+$, $^{3/2}$[**4**]; S = 3/2, E = -2066.44088030

C	-1.79089	2.08790	0.41743
O	-1.26884	3.14233	0.66037
O	-1.81086	1.35873	-0.60612
Fe	-2.74573	-0.39163	-0.84452
N	-1.18150	-1.62256	-0.06301
N	-2.09742	-0.39444	-2.74862
N	-4.27811	-1.55135	-1.56085
N	-3.40035	-0.37521	1.06449
H	-1.41114	-2.59629	-0.23142
H	-2.41489	0.50090	-3.10740
H	-4.02374	-2.51101	-1.34463
H	-3.04895	0.53202	1.37599
C	-0.63766	-0.42468	-2.94091
H	-0.25914	0.54882	-2.60941
H	-0.41804	-0.51759	-4.01376
C	0.07766	-1.52329	-2.17895
H	1.11153	-1.54086	-2.54397
H	-0.33483	-2.51106	-2.42962
C	0.11890	-1.34958	-0.67470
H	0.38789	-0.31540	-0.42441
H	0.89019	-2.00313	-0.24144
C	-1.22771	-1.38119	1.37903
H	-0.63089	-2.11008	1.94416
H	-0.79817	-0.38738	1.56128
C	-2.66936	-1.40112	1.82020
H	-2.75468	-1.23166	2.90177
H	-3.11249	-2.38096	1.60539
C	-4.85069	-0.38726	1.29295
H	-5.05211	-0.48711	2.36879
H	-5.23404	0.59188	0.98128
C	-5.58254	-1.47614	0.53525
H	-5.18399	-2.46735	0.79456
H	-6.62178	-1.47893	0.88452
C	-5.59914	-1.30736	-0.96809
H	-5.89776	-0.28569	-1.23450
H	-6.33546	-1.98954	-1.41513
C	-4.25907	-1.41108	-3.02405
H	-4.85103	-2.19467	-3.51385
H	-4.71575	-0.44500	-3.27120
C	-2.82347	-1.44137	-3.47789
H	-2.74454	-1.30715	-4.56433
H	-2.37411	-2.40987	-3.23024

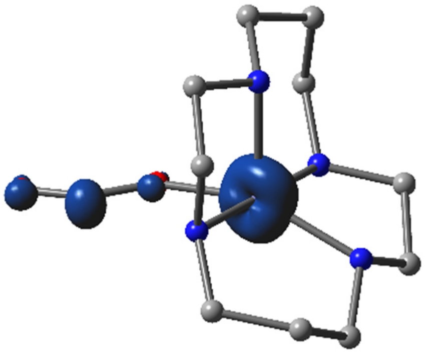

Figure S16. Spin density of $^{3/2}$[**4**].

Atomic charges and spin densities from Natural Population Analysis:

1 C		0.119001	0.683005
2 O		-0.355795	0.156340
3 O		-0.403178	0.103260
4 Fe		0.415554	2.029502

Spin density on the CO_2-ligand: 0.942605

trig.-bipyram.-[Fe(cyclam)(equat.-η_O^1-OCO)]$^+$, $^{5/2}$[**5**]; S = 5/2, E = -2066.45618406

C	-2.62918	2.55168	0.15704
O	-2.56648	3.73794	0.32450
O	-2.44233	1.82116	-0.85409
Fe	-2.65287	-0.16831	-0.79253
N	-1.17774	-1.57409	-0.05080
N	-1.93602	-0.39334	-2.85427
N	-4.19666	-1.49780	-1.55347
N	-3.39609	-0.39475	1.25137
H	-1.47701	-2.52345	-0.24890
H	-2.17806	0.50851	-3.24865
H	-3.93557	-2.46081	-1.36731
H	-3.11060	0.50261	1.63327
C	-0.49126	-0.58170	-3.03490
H	-0.01165	0.37693	-2.80413
H	-0.27008	-0.80629	-4.08926
C	0.11601	-1.66266	-2.15929
H	1.15386	-1.79116	-2.48933
H	-0.36664	-2.63335	-2.34490
C	0.13929	-1.38726	-0.66792
H	0.45699	-0.35420	-0.47361
H	0.87520	-2.04601	-0.18385
C	-1.18572	-1.39749	1.40389
H	-0.57414	-2.15750	1.90961
H	-0.74092	-0.41831	1.62404
C	-2.61035	-1.44392	1.90421
H	-2.63697	-1.34873	2.99833
H	-3.05532	-2.41498	1.65563
C	-4.84609	-0.51477	1.43441
H	-5.07632	-0.73586	2.48757
H	-5.27899	0.46799	1.21087
C	-5.50277	-1.55894	0.55045
H	-5.06502	-2.55216	0.72836
H	-6.54655	-1.64252	0.87626
C	-5.50792	-1.26771	-0.93767
H	-5.78520	-0.22135	-1.12201
H	-6.26574	-1.89292	-1.43222
C	-4.18456	-1.30607	-3.00657
H	-4.82780	-2.03617	-3.51703
H	-4.59069	-0.30834	-3.21639
C	-2.76468	-1.40725	-3.51061
H	-2.73584	-1.30917	-4.60431
H	-2.35864	-2.39619	-3.26653

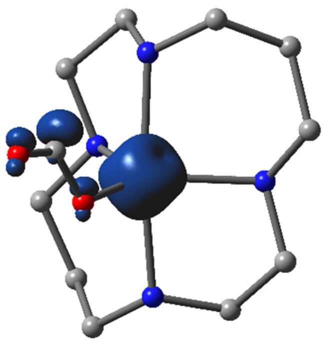

Figure S17. Spin density of $^{5/2}$[**5**].

Atomic charges and spin densities from Natural Population Analysis:

1	C	0.140125	0.708223
2	O	-0.347757	0.153018
3	O	-0.480499	0.133555
4	Fe	0.709915	3.830995

Spin density on the CO_2-ligand: 0.994796

square-pyram.-[Fe(cyclam)(basal-η_O^1-OCO)]$^+$, $^{5/2}$[**4**]; S = 5/2, E = -2066.45707703

C	-3.43810	2.32642	-1.77198
O	-3.89029	3.40876	-2.02563
O	-3.53155	1.57419	-0.76551
Fe	-2.61064	-0.22548	-0.65217
N	-1.19341	-1.76142	-0.01847
N	-1.84394	-0.32786	-2.68957
N	-4.19865	-1.40538	-1.52570
N	-3.31600	-0.52360	1.39408
H	-1.55796	-2.66565	-0.30108
H	-2.04852	0.62443	-2.98221
H	-4.01217	-2.39731	-1.42334
H	-2.98235	0.28929	1.89853
C	-0.41152	-0.56952	-2.88885
H	0.11503	0.33799	-2.57033
H	-0.19735	-0.70773	-3.95928
C	0.13018	-1.75715	-2.11503
H	1.16502	-1.91008	-2.44435
H	-0.40091	-2.67853	-2.39513
C	0.14480	-1.62014	-0.60510
H	0.53522	-0.63639	-0.31310
H	0.81917	-2.37444	-0.17435
C	-1.20104	-1.71796	1.44701
H	-0.65629	-2.56877	1.87842
H	-0.68098	-0.80304	1.75804
C	-2.62930	-1.69739	1.93751
H	-2.66070	-1.71454	3.03531
H	-3.14828	-2.59724	1.58733
C	-4.77908	-0.55153	1.52984
H	-5.05257	-0.81124	2.56361
H	-5.13216	0.47073	1.34835
C	-5.49343	-1.49724	0.57972
H	-5.12210	-2.52569	0.69652
H	-6.53975	-1.53394	0.90623
C	-5.48726	-1.12017	-0.88989
H	-5.68371	-0.04659	-1.00827
H	-6.29255	-1.65803	-1.41178
C	-4.14531	-1.07598	-2.95058
H	-4.82752	-1.70079	-3.54399
H	-4.47326	-0.03345	-3.06252
C	-2.72450	-1.22450	-3.44390
H	-2.66992	-1.02726	-4.52339
H	-2.38734	-2.25652	-3.28874

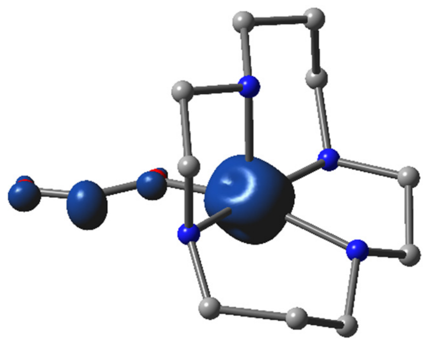

Figure S18. Spin density of $^{5/2}$[**4**].

Atomic charges and spin densities from Natural Population Analysis:

1	C	0.141098	0.702182
2	O	-0.349777	0.154336
3	O	-0.442570	0.123349
4	Fe	0.697283	3.838671

Spin density on the CO_2-ligand: 0.979867

trig.-bipyram.-[Fe(cyclam)(equat.-η_C^1-CO_2)]$^+$,
$^{5/2}$[**6**]; S = 5/2, E = -2066.44789254

C	-2.75937	2.05769	-0.69881
O	-2.86188	2.47712	0.44200
O	-2.67750	2.50924	-1.83021
Fe	-2.71571	-0.24062	-0.78159
N	-1.20579	-1.59166	-0.06214
N	-2.00193	-0.39189	-2.82880
N	-4.24934	-1.54324	-1.54363
N	-3.42509	-0.45486	1.26172
H	-1.50310	-2.54131	-0.26117
H	-2.25723	0.53412	-3.15941
H	-3.97031	-2.50606	-1.38618
H	-3.14808	0.45655	1.61556
C	-0.55645	-0.55854	-3.02958
H	-0.08680	0.40119	-2.78350
H	-0.35064	-0.75473	-4.09183
C	0.06904	-1.65209	-2.18425
H	1.10354	-1.76889	-2.52824
H	-0.41231	-2.62113	-2.38089
C	0.10659	-1.39786	-0.68944
H	0.42807	-0.36854	-0.48292
H	0.84302	-2.06643	-0.22076
C	-1.19772	-1.41907	1.39378
H	-0.57061	-2.17459	1.88625
H	-0.76170	-0.43560	1.61124
C	-2.61556	-1.48730	1.91260
H	-2.63015	-1.38088	3.00549
H	-3.04883	-2.46622	1.67594
C	-4.87459	-0.58945	1.45415
H	-5.09029	-0.80671	2.51032
H	-5.31725	0.38873	1.23039
C	-5.52510	-1.64461	0.57853
H	-5.06642	-2.62954	0.74822
H	-6.56221	-1.74687	0.91941
C	-5.55811	-1.34853	-0.90901
H	-5.86297	-0.30854	-1.08506
H	-6.30549	-1.99132	-1.39601
C	-4.25200	-1.30371	-2.99073
H	-4.89672	-2.02085	-3.51685
H	-4.66415	-0.30120	-3.16278
C	-2.83622	-1.38364	-3.51287
H	-2.81962	-1.23959	-4.60135
H	-2.42401	-2.37935	-3.31134

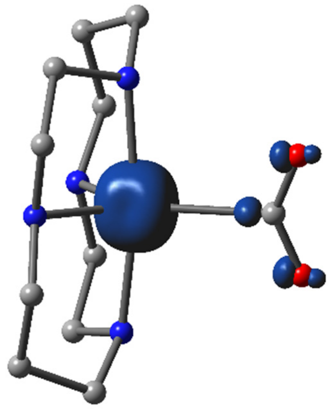

Figure S19. Spin density of $^{5/2}$[**6**].

Atomic charges and spin densities from Natural Population Analysis:

1	C	0.165834	0.412644
2	O	-0.416029	0.149416
3	O	-0.411706	0.159757
4	Fe	0.563858	4.084466

Spin density on the CO_2-ligand: 0.721817

13. References

[1] M. J. Frisch, G. W. Trucks, H. B. Schlegel, G. E. Scuseria, M. A. Robb, J. R. Cheeseman, G. Scalmani, V. Barone, G. A. Petersson, H. Nakatsuji, X. Li, M. Caricato, A. V. Marenich, J. Bloino, B. G. Janesko, R. Gomperts, B. Mennucci, H. P. Hratchian, J. V. Ortiz, A. F. Izmaylov, J. L. Sonnenberg, Williams, F. Ding, F. Lipparini, F. Egidi, J. Goings, B. Peng, A. Petrone, T. Henderson, D. Ranasinghe, V. G. Zakrzewski, J. Gao, N. Rega, G. Zheng, W. Liang, M. Hada, M. Ehara, K. Toyota, R. Fukuda, J. Hasegawa, M. Ishida, T. Nakajima, Y. Honda, O. Kitao, H. Nakai, T. Vreven, K. Throssell, J. A. Montgomery Jr., J. E. Peralta, F. Ogliaro, M. J. Bearpark, J. J. Heyd, E. N. Brothers, K. N. Kudin, V. N. Staroverov, T. A. Keith, R. Kobayashi, J. Normand, K. Raghavachari, A. P. Rendell, J. C. Burant, S. S. Iyengar, J. Tomasi, M. Cossi, J. M. Millam, M. Klene, C. Adamo, R. Cammi, J. W. Ochterski, R. L. Martin, K. Morokuma, O. Farkas, J. B. Foresman, D. J. Fox, Gaussian, Inc., Wallingford, CT, **2016**.

[2] F. Weigend, R. Ahlrichs, *Phys. Chem. Chem. Phys.* **2005**, *7*, 3297-3305.

[3] a) B. I. Dunlap, *J. Chem. Phys.* **1983**, *78*, 3140-3142; b) B. I. Dunlap, *J. Mol. Struct. Theochem.* **2000**, *529*, 37-40.

[4] F. Weigend, *Phys. Chem. Chem. Phys.* **2006**, *8*, 1057-1065.

[5] Y. Zhao, D. G. Truhlar, *J. Phys. Chem. A* **2004**, *108*, 6908-6918.

[6] Y. Zhao, D. G. Truhlar, *J. Phys. Chem. A* **2005**, *109*, 5656-5667.

[7] a) V. Barone, M. Cossi, *J. Phys.Chem. A* **1998**, *102*, 1995-2001; b) M. Cossi, N. Rega, G. Scalmani, V. Barone, *J. Comp. Chem.* **2003**, *24*, 669-681.

[8] S. Grimme, S. Ehrlich, L. Goerigk, *J. Comp. Chem.* **2011**, *32*, 1456-1465.

[9] S. Hirata, M. Head-Gordon, *Chem. Phys. Lett.* **1999**, *314*, 291-299.

[10] a) J. D. Chai, M. Head-Gordon, *Phys. Chem. Chem. Phys.* **2008**, *10*, 6615-6620; b) J. D. Chai, M. Head-Gordon, *J. Chem. Phys.* **2008**, *128*.

[11] R. L. Martin, *J. Chem. Phys.* **2003**, *118*, 4775-4777.

[12] a) S. Straub, L. I. Domenianni, J. Lindner, P. Vöhringer, *J. Phys. Chem. B* **2019**, *123*, 7893-7904; b) S. Straub, P. Brünker, J. Lindner, P. Vöhringer, *Phys. Chem. Chem. Phys.* **2018**, *20*, 21390-21403; c) B. Wezisla, J. Lindner, U. Das, A. C. Filippou, P. Vöhringer, *Angew. Chem. Int. Ed.* **2017**, *56*, 6901-6905.

[13] P. K. Chan, C. K. Poon, *J. Chem. Soc. Dalton Trans.* **1976**, 858-862.

[14] D. Broadbent, J. Dollimore, D. Dollimore, T. A. Evans, *J. Chem. Soc. Faraday Trans.* **1991**, *87*, 161-166.

[15] J. F. Berry, F. A. Cotton, T. B. Lu, C. A. Murillo, *Inorg. Chem.* **2003**, *42*, 4425-4430.

[16] a) A. D. Becke, *Phys. Rev. A* **1988**, *38*, 3098-3100; b) J. P. Perdew, *Phys. Rev. B* **1986**, *33*, 8822-8824.

[17] S. Grimme, *J. Comp. Chem.* **2006**, *27*, 1787-1799.

[18] M. Swart, A. W. Ehlers, K. Lammertsma, *Mol. Phys.* **2004**, *102*, 2467-2474.

[19] Y. Zhao, D. G. Truhlar, *J. Chem. Phys.* **2006**, *125*.

[20] J. M. Tao, J. P. Perdew, V. N. Staroverov, G. E. Scuseria, *Phys. Rev. Lett.* **2003**, *91*.

[21] a) A. D. Becke, *J. Chem. Phys.* **1993**, *98*, 5648-5652; b) M. Reiher, O. Salomon, B. A. Hess, *Theoret. Chem. Acc.* **2001**, *107*, 48-55; c) O. Salomon, M. Reiher, B. A. Hess, *J. Chem. Phys.* **2002**, *117*, 4729-4737; d) S. H. Vosko, L. Wilk, M. Nusair, *Can. J. Phys.* **1980**, *58*, 1200-1211; e) C. T. Lee, W. T. Yang, R. G. Parr, *Phys. Rev. B* **1988**, *37*, 785-789.

[22] P. J. Stephens, F. J. Devlin, C. F. Chabalowski, M. J. Frisch, *J. Phys. Chem.* **1994**, *98*, 11623-11627.

[23] C. Adamo, V. Barone, *Chem. Phys. Lett.* **1997**, *274*, 242-250.

[24] C. Adamo, V. Barone, *J. Chem. Phys.* **1999**, *110*, 6158-6170.

[25] M. Ernzerhof, J. P. Perdew, *J. Chem. Phys.* **1998**, *109*, 3313-3320.

[26] A. D. Becke, *J. Chem. Phys.* **1993**, *98*, 1372-1377.

[27] a) V. N. Staroverov, G. E. Scuseria, J. M. Tao, J. P. Perdew, *J. Chem. Phys.* **2003**, *119*, 12129-12137; b) V. N. Staroverov, G. E. Scuseria, J. M. Tao, J. P. Perdew, *J. Chem. Phys.* **2004**, *121*, 11507-11507.

[28] E. Brémond, C. Adamo, *J. Chem. Phys.* **2011**, *135*.
[29] L. Goerigk, S. Grimme, *J. Chem. Theory Comput.* **2011**, *7*, 291-309.
[30] a) S. Kozuch, J. M. L. Martin, *Phys. Chem. Chem. Phys.* **2011**, *13*, 20104-20107; b) S. Kozuch, J. M. L. Martin, *J. Comp. Chem.* **2013**, *34*, 2327-2344.
